# Single cell analysis reveals the involvement of the long non-coding RNA Pvt1 in the modulation of muscle atrophy and mitochondrial network

**DOI:** 10.1093/nar/gkz007

**Published:** 2019-01-16

**Authors:** Enrico Alessio, Lisa Buson, Francesco Chemello, Caterina Peggion, Francesca Grespi, Paolo Martini, Maria L Massimino, Beniamina Pacchioni, Caterina Millino, Chiara Romualdi, Alessandro Bertoli, Luca Scorrano, Gerolamo Lanfranchi, Stefano Cagnin

**Affiliations:** 1 Department of Biology, University of Padova, 35131 Padova, Italy; 2 Department of Biomedical Sciences, University of Padova, 35131 Padova, Italy; 3 CNR Neuroscience Institute, 35131 Padova, Italy; 4 CRIBI Biotechnology Center, University of Padova, 35131 Padova, Italy; 5 Padova Neuroscience Center, University of Padova, 35131 Padova, Italy; 6 Venetian Institute of Molecular Medicine, 35131 Padova, Italy; 7 CIR-Myo Myology Center, University of Padova, 35131 Padova, Italy

## Abstract

Long non-coding RNAs (lncRNAs) are emerging as important players in the regulation of several aspects of cellular biology. For a better comprehension of their function, it is fundamental to determine their tissue or cell specificity and to identify their subcellular localization. In fact, the activity of lncRNAs may vary according to cell and tissue specificity and subcellular compartmentalization. Myofibers are the smallest complete contractile system of skeletal muscle influencing its contraction velocity and metabolism. How lncRNAs are expressed in different myofibers, participate in metabolism regulation and muscle atrophy or how they are compartmentalized within a single myofiber is still unknown. We compiled a comprehensive catalog of lncRNAs expressed in skeletal muscle, associating the fiber-type specificity and subcellular location to each of them, and demonstrating that many lncRNAs can be involved in the biological processes de-regulated during muscle atrophy. We demonstrated that the lncRNA Pvt1, activated early during muscle atrophy, impacts mitochondrial respiration and morphology and affects mito/autophagy, apoptosis and myofiber size *in vivo*. This work corroborates the importance of lncRNAs in the regulation of metabolism and neuromuscular pathologies and offers a valuable resource to study the metabolism in single cells characterized by pronounced plasticity.

## INTRODUCTION

Long non-coding RNAs (lncRNAs) are transcripts longer than 200 nucleotides, without protein coding potential, encompassing ∼144 000 and 126 000 loci in human and mouse genomes, respectively (http://www.noncode.org/). Recently, the complete sequence of 28 000 human lncRNA genes with their genuine 5′ transcriptional start site was defined (1). However, future expression studies at higher definition will likely increase this number as lncRNAs are more tissue-, cell-specific and less expressed than messenger RNA (mRNAs) (2–4). The mechanisms of action of most annotated lncRNAs are still unknown. The new frontier of single cell analysis will surely improve our knowledge of cell-specific and low abundant lncRNAs (5). A great advancement in the comprehension of lncRNA functional roles could be provided by associating their expression at single cell level with subcellular localization. Recently, it was demonstrated that most lncRNAs are strongly enriched in the cytosol and in ribosomal fractions (6), thus contributing to regulate microRNA (miRNA) activity (7).

One of the most abundant tissues in vertebrates is skeletal muscle. It is responsible for motor activity and significantly contributes to whole-body metabolism (8). In fact, skeletal muscle takes up glucose from blood to maintain glucose homeostasis and controls body heat for core temperature maintenance. Furthermore, during fasting, muscle can rapidly adapt to use fatty acids and amino acids as oxidative substrates. Skeletal muscle is a heterogeneous tissue composed by different cell types interspersed among an ordered array of myofibers, which are the smallest complete contractile system responsible for muscle metabolic and contractile traits. Myofibers are also physiologically heterogeneous. In fact, human skeletal muscles are mainly composed by three types of myofibers: slow-twitch type 1, that are mitochondria-rich and rely on oxidative metabolism; oxidative fast-twitch type 2a and glycolytic type 2x (9). In mice, there are also type 2b myofibers that are glycolytic fast-twitch myofibers. Myofiber type, number and cross-sectional area are affected in pathophysiological conditions (10). Such plasticity is modulated by a series of sensor proteins that are mobilized from the cytoplasm to nuclear chromatin (11–13), and by mitochondrial dynamics. In fact, mitochondria are elongated due to increased fusion in oxidative myofibers compared to glycolytic myofibers (14). If and how lncRNAs participate in the myofiber specification and physiology, is currently under investigation.

In this study, we provide a complete catalog of lncRNAs, based on the Ensembl 74 database, that are expressed by slow-oxidative and fast-glycolytic myofibers. We characterized their subcellular localization evidencing that most of them are cytoplasmic.

Some of the lncRNAs we detected in myofibers are involved in different pathologies of skeletal muscle that cause muscle atrophy through the induction of mitochondrial fragmentation, apoptotic process and autophagy. Among the fiber-specific lncRNAs we identified, we functionally characterized the lncRNA Pvt1 showing its ability to modulate skeletal muscle atrophy. We demonstrated that Pvt1 function in skeletal muscle is related to the direct regulation of c-Myc stability that modulates the expression of Bcl-2, Bax/Bak, Mfn1 and Beclin 1. Bcl-2 is regulated by c-Myc and represents a central node in the regulation of autophagy and apoptosis, that is induced by mitochondrial fragmentation (15,16). The *in vivo* modulation of Pvt1 expression results in the attenuation of mitochondrial fragmentation, apoptosis, autophagy and myofiber atrophy. The induction of mitochondrial fusion, promoted by Pvt1 down-regulation, is also associated with an increased production of adenosine triphosphate (ATP) in muscle cells. Overall, our data represent a valuable resource to study metabolism in single cells characterized by a pronounced plasticity and corroborate the importance of lncRNAs in the regulation of muscle metabolism and neuromuscular pathologies.

## MATERIALS AND METHODS

### Mouse models

Mice were housed in individual cages in an environmentally controlled room (23°C, 12 h light-dark cycle) and provided with food and water *ad libitum*.

Two models of muscle wasting were used: amyotrophic lateral sclerosis (ALS), and denervation. The ALS mouse model consisted of transgenic mice expressing the ALS-related human SOD1 gene (B6SJL(Tg-SOD1*G93A)1Gur/J, The Jackson Laboratories; (17)). The colony was maintained by breeding hemizygote transgenic males with wild-type B6SJLF1/J hybrid females. Newborn animals were genotyped using standard procedures for identifying transgenic individuals, which develop a motor neuron disease resembling ALS features. Male mice were sacrificed at 1 (presymptomatic), 3 (symptomatic) or 4 (terminally-ill) months of age, and transgenic age-matched mice expressing WT human SOD1 gene (B6SJL-Tg(SOD1)2Gur/J, The Jackson Laboratories) were used as control.

Denervation was performed on adult, 2-month-old male CD1 mice (Charles River). Denervation experiments were performed by cutting the sciatic nerve of one limb while leaving the nerve intact in the contralateral limb of the same animal to be used as control. A pool of RNA samples extracted from non-denervated limbs was used as control. Mice were sacrificed at day 3, 7 or 14 after denervation. CD1 mice were also used to alter the expression of Pvt1 *in vivo*, for subcellular localization by fluorescence in situ hybridization (FISH) and the genome-wide approach, and to isolate myofibers for the identification of differences in the expression of lncRNAs between slow type 1 and fast type 2b myofibers. For these last experiments, female mice were sacrificed by rapid cervical dislocation at month 3 of age. All experimental procedures and animal care protocols performed on SOD1(G93A) mice, the control counterpart and CD1 mice were approved by the Italian Ministry of Health (authorization N. 305/2017-PR), and by the Ethical Committee for animal care and use of the University of Padova (OPBA). All efforts were made to minimize animal suffering.

### Cell cultures

C2C12 myoblasts were cultured on Tissue Culture Dishes or Tissue Culture Multiwell Plates (Thermo Fisher Scientific). Myoblasts were maintained in proliferation medium (Dulbecco's modified Eagle’s medium (DMEM), 10% foetal bovine serum, 1 unit/ml Penicillin, 100 μg/ml Streptomycin), detached when reaching ∼80% confluence using Trypsin-ethylenediaminetetraacetic acid (Thermo Fisher Scientific) and re-seeded at a lower density.

Differentiation was induced by switching from proliferation medium to differentiation medium (DMEM, 2% horse serum, 1 unit/ml Penicillin, 100 μg/ml Streptomycin) upon reaching confluence and protracted until 1, 3, 7 or 14 days.

Mouse primary myoblasts were acquired by Applied Biological Materials (abm) Inc. (Canada) and ∼3500 cells/cm^2^ were seeded in PriCoat™ T25 Flasks using the Prigrow X series medium and 1% Penicillin-Streptomycin as suggested by the company. All cells were maintained in humidified incubator at CO_2_ 5% and 37°C.

### Nucleus–cytoplasm fractionation

To separate nuclei from cytoplasm, myofibers from *extensor digitorum longus* (EDL), *soleus* or *tibialis anterior* (TA) were purified and classified according to the protocol described in (18–20) and then 5–10 myofibers were pooled. C2C12 cultures were, instead, washed in phosphate-buffered saline (PBS) and detached from 10 cm Tissue Culture Plate using a cell scraper and 280 μl of RLN Buffer (Water, 50 mM Tris–HCl pH 8.0, 140 mM NaCl, 1.5 mM MgCl_2_, 0.5% v/v Triton-X100, 0.36 units/μl RNase OUT). Cells were moved into a microcentrifuge tube and lysis was performed by repeatedly passing the solution through a 0.2 μm needle. The RLN buffer was also used to lyse pooled myofibers through the same type of needle.

To check that the lysis process was completed and that all nuclei were separated from the myofibers, a small aliquot of the solution was stained with SYBR safe (Life Technologies) and then observed at the fluorescent and bright field microscope (Supplementary Figure S1).

Nuclei were then pelleted in a microcentrifuge for 5 min at 600 × g at 4°C. Supernatants (cytoplasmic fractions) were moved to different tubes and their volumes were reduced by 50% in a Savant SpeedVac concentrator. A further control of the purity of nuclei and cytoplasmic preparations was performed on RNA extracted from the two subcellular fractions basing on observations made in (21,22) (Supplementary Figure S1). Agilent 2100 Bioanalyzer and RNA nano chips were used according to the protocol of the manufacturer.

### RNA extraction

TRIzol Reagent (Thermo Fisher Scientific) was used to extract RNA from C2C12 and satellite cell cultures (1 ml every 65 cm^2^ of growth surface), whole muscle tissue (1 ml every 30 μg of tissue), single myofibers (500 ul per myofiber) and from purified nuclei or cytoplasm (1 ml for every fraction). RNA extraction from single myofibers was previously described (18–20).

Prior to RNA extraction, C2C12 and satellite cell cultures were washed with PBS to remove excess of medium. Then, TRIzol was added directly on the dish and cells were detached using a cell scraper. Tissue biopsies and single myofibers were immersed in TRIzol shortly after excision. Biopsies were homogenized using a TissueLyser II (QIAGEN) while single myofibers and nuclei were lysed by pipetting the solution. TRIzol was also used to extract RNA from cytoplasm. RNA quality was tested on UV spectrophotometer and 2100 Agilent Bioanalyzer following the protocol provided by the manufacturer and only samples with RIN higher than 7.5 were used for following experiments.

### Genome wide analysis of lncRNA expression and subcellular localization in skeletal muscle myofibers

RNAs extracted from single fast and slow myofibers, such as that from nuclei and cytoplasm of skeletal muscle myofibers, were analyzed using a microarray chip.

We started from the probe sequences included in the SurePrint G3 Mouse Gene Expression 8 × 60K Agilent chip and re-annotated the sequence probes according to their ability to bind lncRNAs included in the Ensembl 74 database (GPL24842).

Microarray experiments were performed on single myofibers to dissect the association of myofibers metabolism with lncRNA expression and on pools of 5–10 myofibers obtained from EDL, *Soleus* and TA to dissect preferential subcellular localization of lncRNAs. Microarray experiments were performed on a total of 21 myofibers purified from nine mice while subcellular localization experiments were replicated using different pools of fibers from the same types of muscles of nine wild-type CD1 mice. Quantitative real time PCR (qPCR) in association with myofibers purification and nuclei-cytoplasm cell fractionation were also used to confirm the myofiber specific expression of different lncRNAs and to dissect subcellular localization of specific lncRNAs.

### Microarray experiments and data analysis

Fluorescent chromosomal RNA to hybridize onto microarray was produced by Low Input Quick Amp Labelling Kit (Agilent) according to manufacturer instructions. We used a different approach when experiments were performed on single myofibers. To obtain enough complementary DNA (cDNA) for microarray experiments, RNA purified from a single myofiber was amplified using the TransPlex Whole Transcriptome Amplification 2 Kit (Sigma-Aldrich) in accordance with the instructions of the manufacturer. Labeling was performed by Genomic DNA Enzymatic Labelling Kit (Agilent Technologies) as described by the manufacturer.

Labeled sample was dispensed onto the microarray to perform hybridization at 65°C for 17 h with 10 rpm rotation. Finally, slides were washed using Wash Buffer Kit (Agilent Technologies) and dried at room temperature (for extended protocol see Supplementary Methods).

Microarray slides were scanned using G2505C scanner (Agilent Technologies) at 3 μm resolution. Probes features were extracted using the Feature Extraction Software v. 10.7.3.1 with GE_1_Sep09 protocol (Agilent Technologies). Intra-array normalization was directly performed by the Feature Extraction Software. The raw data are available in the GEO database (single myofiber lncRNA expression analysis: GSE112716; nuclear-cytoplasmic lncRNA localization: GSE112768; Pvt1 down-expression: GSE112881). For each sample, we set probe expression to NA (not available) when the flag ‘Positive and Significant’ from Feature Extraction Software was ‘FALSE’. To normalize data, we used quantile inter-arrays normalization (normalizeQuantiles, limma R package). The expression of probes with the same ProbeName was averaged.

Microarray data were analyzed using the MultiExperiment Viewer (MeV, Ver. 4.8) (23). We used a *t*-test (from MeV) to identify differentially expressed lncRNAs between fast and slow fiber types. *P*-values were computed using a gene permutation approach and corrected using Bonferroni. We considered a gene differentially expressed when corrected *P*-value was ≤5 × 10^−2^.

Hierarchical clustering on myofibers was performed using Pearson’s correlation distance and complete linkage method. To identify subcellular location in which lncRNAs were preferentially expressed, normalized data were analyzed with Statistical Analysis of Microarray. The delta value was chosen to maximize the number of significant genes while keeping the median number of false positives under 1%. To infer the function of coding genes differentially expressed after Pvt1 down-expression, we grouped them according to gene ontology using the WEB-based GEne SeT AnaLysis Toolkit (24).

### Quantitative real time PCR analysis

Extracted RNA was retrotranscribed into cDNA using SuperScript II Reverse Transcriptase (Thermo Fisher Scientific) and gene expression was evaluated in a 7500 Real-Time polymerase chain reaction (PCR) System (Applied Biosystems), using the EvaGreen chemistry (Solis ByoDyne) (for the extended protocol see Supplementary Methods). Thermocycler was set as follow: activation step (×1) 95°C for 12 min; PCR Cycle (×40) 95°C for 15 s. (denaturation), 60°C for 20 s. (annealing), 72°C for 35 s. (elongation); final elongation (×1) 72°C for 3 min; dissociation curve (×1). Original expression level was calculated as 2−[*C*t gene of interest – *C*t House Keeping Gene] and then normalized according to the average of the expression of the gene in all samples. In the analysis of single myofibers we used three technical replicates for each myofibers while in other conditions we used at least three biological and two technical replicates. The reference genes used were TATA box binding protein (Tbp), Thioredoxin 1 (Txn1), or β-2 microglobulin (B2m). Reference genes were chosen according to our previous studies on single myofibers (18–20) and their homogeneous expression in analyzed samples. The list of primers used for qPCR is included in the Supplementary Table S1.

### Fluorescence in situ hybridization (FISH)

#### Probes construction

PCR on the cDNA was used to produce an amplicon of ∼600 base pairs from the chosen lncRNAs (see Supplementary Table S2 for the list of primers). Amplicons were cloned into pSC-A-amp/kan plasmid using StrataClone PCR Cloning Kit (Agilent Technologies). Plasmids were amplified, purified with the PureLink MiniPrep kit (Thermo Fisher Scientific) and the sequences were checked by Sanger sequencing (Supplementary Table S2). Plasmids were linearized using a restriction enzyme (HindIII or SmaI; New Englend Biolabs) to allow *in vitro* transcription for the production of the FISH probes.

#### FISH experiment

FISH experiments were performed on both proliferating and differentiating C2C12 cells and on 20 μm thick sections of TA (for extended protocol see Supplementary Methods).

#### Image acquisition and analysis

Images were acquired with Leica TCS SP5 confocal laser microscope. When comparing fluorescence levels was necessary, images were acquired during the same session, using the same microscope parameters.

### Pvt1 down-expression

#### In vitro gene expression modulation

*In vitro* experiments to down-express Pvt1 were performed by transfecting proliferating C2C12 myoblasts with Lipofectamine 2000 Transfection Reagent (Termo Fisher Scientific) and antisense LNA GapmeRs (Exiqon) (Pvt1_1 ACCGTAGTAGAGTTAA; Pvt1_3 AGTCAACGCTTCACAT). Cells transfected with Lipofectamine 2000 and Antisense LNA GapmeR Negative Controls (Exiqon) were used as negative controls. Silencing efficiency was assessed by qPCR analysis (for extended protocol see Supplementary Methods).

#### In vivo Pvt1 expression modulation

*In vivo* experiments to down-express Pvt1 were performed on healthy and denervated CD1 mice using the same GapmeRs described for *in vitro* silencing. Invivofectamine 3.0 Reagent (Thermo Fisher Scientific) was used as transfecting reagent through the injection in one limb of 100 μl transfecting solution (Complexation Buffer, Invivofectamine, GapmeRs).

Three mice for each group were treated with 1.9 nmol of Pvt1 specific GapmeRs while the other three were treated with Negative Control GapmeRs at the same concentration. Multiple injections were performed on both sides of the treated limb to make sure that all muscles of the limb were transfected. All mice were treated a second time, 2 days after the first treatment. Muscles from both limbs of each mouse were collected 4 days after the second treatment. Silencing efficiency was assessed on TA muscles analyzing the expression of Pvt1 by qPCR.

### Flow cytometry

After Pvt1 down-expression, C2C12 myoblasts were permeabilized and stained for 15 min with BODIPY 493/503 (2 μg/ml, Life Technologies). After two PBS washes, cells were transferred in FACS conical tubes and flow cytometry was performed using a BD FACSCalibur platform (Becton Dickinson). Percentage of positive cells was calculated using mock-transfected C2C12 as a negative control. Results derive from the average of three independent experiments.

### Skeletal muscle cryosections and ultrastructural analyses

#### Muscle cryosection analysis

Succinate dehydrogenase stain was performed incubating fresh muscle cryosections derived from *gastrocnemius* for 30 min as described in (25). Periodic acid-Schiff (PAS) staining was performed on cryosections of *gastrocnemius* following the instructions of PAS staining system (Sigma-Aldrich). Images were acquired with a 10× objective using Leica DM R microscope. Images were processed using ImageJ software.

#### Electron microscopy analysis

TA were fixed with 2.5% glutaraldehyde in 0.1 M sodium cacodylate buffer pH 7.4 for 1 h at 4°C, postfixed with 1% osmium tetroxide and 1% in 0.1 M sodium cacodylate buffer for 2 h at 4°C. After three water washes, samples were dehydrated in a graded ethanol series and embedded in an epoxy resin (Sigma-Aldrich). Ultrathin sections (60–70 nm) were obtained with an Ultrotome V (LKB) ultramicrotome, counterstained with uranyl acetate and lead citrate and viewed with a Tecnai G2 (FEI) transmission electron microscope operating at 100 kV. Images were captured with a Veleta (Olympus Soft Imaging System) digital camera.

### Mitochondrial respiration

Control cells transfected with control GapmeRs and C2C12 cells where Pvt1 was down-expressed were cultured as previously reported. Mitochondrial respiration was investigated by using the Mitochondrial ToxGlo™Assay (Promega) according to instructions of the manufacturer. After 48 h of transfection, cells were cultured into galactose and antibiotics-free medium to improve mitochondrial responsiveness. Oligomycin was chosen as mitochondrial toxin at the starting concentration of 25 μM. Dimethyl sulfoxide (DMSO) was used as matched vehicle control. Serial dilutions of mitochondrial toxin were repeated until 1.9 μM. Drug treatment was carried out for 2 h. Luminescence was measured with Infinite 200 Pro plate reader (Tecan i-control) on a bottom clear 384-well plate (Corning).

### Mitochondrial network and mass analysis

C2C12 cell cultures were transfected with mito-RFP vector to stain mitochondria simultaneously with the Pvt1 silencing. Transfections were performed with lipofectamine 2.0 (Life Technologies) and 300 ng of mito-RFP vector. Cells were seeded on coverslips and after 48h of transfection were washed with PBS and fixed with paraformaldehyde 4% for 15 min. After three PBS washes, the coverslips were incubated with BODIPY 493/503 2 μg/ml (Life Technologies) and washed with PBS one more time before mounting the coverslips on glass slides using Fluoromount (Sigma-Aldrich) as mounting medium. Each transfection was replicated independently five times. Images were acquired with a 60× objective using a confocal spinning-disk microscope (Andromeda iMIC system; TILL Photonics). Z-stacks images of ten randomly chosen fields for each coverslip were acquired and stored for subsequent analysis. Images were processed using ImageJ software. Mitochondrial morphology was measured using MitoLoc software (26) and f-index was used to describe mitochondrial network. The f-index is defined as the sum of relative fragment volumes that individually constitute <20% of the total mitochondrial volume. Mitochondrial mass was calculated quantifying mtDNA content in relationship to genomic DNA. DNA was purified by a DNA isolation Kit (Qiagen).

### Western blot analysis

After Pvt1 down-expression, C2C12 cells were washed with PBS and then lysed using a non-reducing buffer containing 10% (v/v) glycerol, 2.3% (v/v) sodium dodecyl sulphate (SDS), 62.5 mM Tris–HCl (pH 6.8) and phosphatase and protease inhibitor cocktails (PhosSTOP and cOmplete mini tables, Roche). Samples were centrifuged at 16 000 × g (10 min, 4°C) to remove cell debris and the protein-containing supernatants were then stored at −80°C. Protein quantification was performed using the Pierce BCA protein assay kit (Pierce, Thermo Scientific) according to manufacturer’s instruction. C2C12 lysates were then adjusted to an equal protein concentration using reducing (50 mM dithiothreitol) Laemmli sample buffer and boiled for at least 5 min. Proteins (20 μg) were separated by sodium dodecylsulphate-polyacrylamide gel electrophoresis using Mini-PROTEAN TGX precast gels (4–15% acrylamide concentration, Bio-Rad Laboratories) and then electroblotted onto 0.22 μm-pore size polyvinylidene difluoride membranes (PVDF, Bio-Rad Laboratories). PVDF membranes were incubated (1 h, room temperature) with a blocking solution [Tris-buffered saline added with 0.1% (w/v) Tween-20 (TBS-T) with either 5% (w/v) instant non-fat dry milk (First Street) or 3% (w/v) bovine serum albumin (BSA) (AppliChem)] followed by incubation with the primary antibody (overnight, 4°C, see below). After three washes (5 min each in TBS-T), membranes were incubated (1 h, room temperature) with a horseradish peroxidase-conjugated anti-rabbit-IgG, anti-mouse-IgG secondary or anti-chicken IgY-secondary antibody, depending on the used primary antibody. After three washes (5 min each in TBS-T), immunoreactive bands were visualized and digitalized with the Alliance Mini HD9 UVITEC imaging system (Eppendorf), using the chemiluminescent HRP substrate reagent kit (EMD Millipore). To verify equal loading and transfer, PVDF membranes were stained with Coomassie brilliant Blue (0.1% w/v Brilliant Blu R (Sigma), 50% methanol, 7% acetic acid). For densitometric analysis, the intensity of each immunoreactive band was normalized to the optical density of the corresponding Coomassie blue-stained lane as in (27–31).

#### Antibodies

Primary antibodies used in western blot were (Ab code and dilution in parenthesis): rabbit monoclonal anti-Beclin1 (D40C5) (1:1000, 3% BSA in 0.1% TBS-T, Cell Signaling Technology); rabbit polyclonal anti-Bax (Δ 21) (SC6236) (1:1000, 1% BSA in 0.1% TBS-T, Santa Cruz Biotechnology); rabbit polyclonal anti-Bak (06–536) (1:1000, 1% BSA in 0.1% TBS-T, Merck-Millipore); rabbit polyclonal anti-Bcl2 (SAB4500003) (1:1000, 1% BSA in 0.1% TBS-T, Sigma-Aldrich); rabbit polyclonal anti-phospho-c-Myc (M8433) (pThr^58^/pSer^62^) (1 μg/ml, 1% BSA in 0.1% TBS-T, Sigma-Aldrich); chicken polyclonal anti-c-Myc (GW21184P) (1:5000, 1% BSA in 0.1% TBS-T, Sigma-Aldrich); rabbit polyclonal anti-Mfn1 (H65) (1:5Z00, 5% non-fat dry milk in 0.1% TBS-T, Santa Cruz Biotechnology).

Secondary antibodies used were: goat anti-mouse IgG-HRP conjugated (SC2005) (1:3000, 1% BSA in 0.1% TBS-T, Santa Cruz Biotechnology); goat anti-chicken IgY-HRP conjugated (SC2428) (1:3000, 1% BSA in 0.1% TBS-T, Santa Cruz Biotechnology); goat anti-rabbit IgG-HRP conjugated (A0545) (1:60 000, 1% BSA in 0.1% TBS-T, Sigma-Aldrich).

## RESULTS

### Expression of lncRNAs in single skeletal muscle fibers

**Figure 1.**
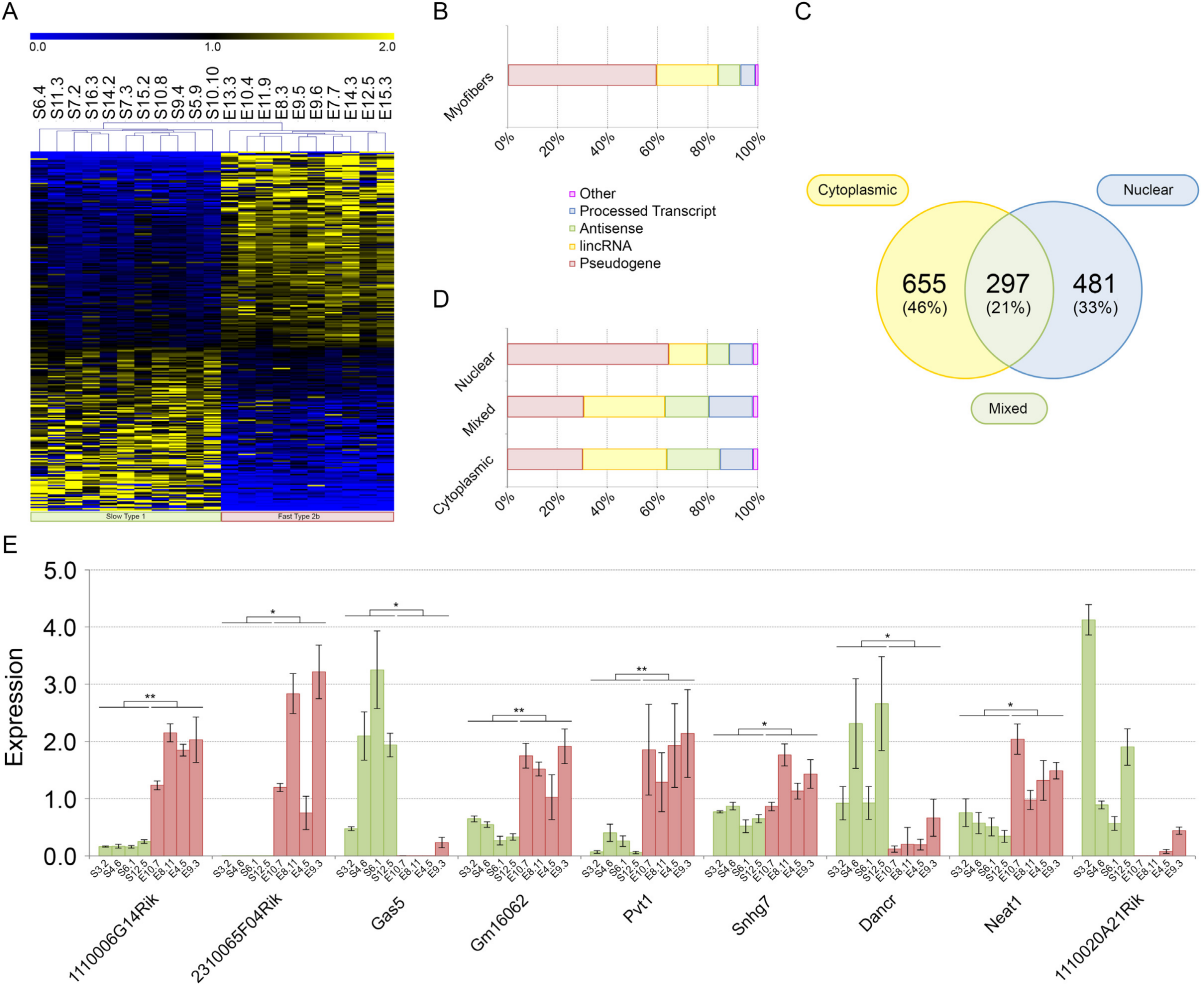
Genome wide analysis of lncRNAs. (**A**) Heat map of lncRNA expression in single myofibers. Initials for myofibers indicate: the muscle source (S = *soleus*; E = EDL), the myofiber number and the mouse number (separated by a dot). (**B**) Categorization of differentially expressed lncRNAs between fast and slow myofibers. About 80% were pseudogenes and lincRNAs. (**C**) Subcellular localization of lncRNAs extracted from nuclear or cytoplasmic fractions of myofibers from *soleus*, EDL and TA. A total of 46% of lncRNAs were cytoplasmic, 33% nuclear and 21% showed a specific localization that changes in relation to the muscle source or lncRNA isoform. (**D**) Categorization of lncRNAs localized in the nucleous or in the cytoplasm of myofibers purified from *soleus*, EDL and TA. Most lncRNAs with cytoplasmic or mixed localization were pseudogenes (∼30%) or (lincRNAs; ∼33%). Nuclear fraction was enriched with pseudogenes while lincRNAs represented ∼15% and antisense ∼9%. (**E**) qPCR for nine lncRNAs. Histograms represent expression value relative to the average expression of the gene among samples. Standard deviation for three technical replicates is indicated. Txn1 was used as control gene. Symbols for myofibers are as described in (A) such as color coding for fast and slow myofibers. The statistical significance between the two groups of myofibers was computed using analysis of variance Student *t*-test for two-tailed distribution and unequal variance. * *P* ≤ 5 × 10^−2^, ** *P* ≤ 1 × 10^−2^.

Different myofibers have different characteristics. For example, metabolically, skeletal muscle fibers can be divided in glycolytic and oxidative with mitochondrial dynamic that is tailored with myofiber diversity (14). The expression of lncRNAs is more tissue- and cell-type specific than that of coding RNAs (32) and this is a solid clue for them being important player in the specification of myofiber characteristics. Before analyzing the expression of lncRNAs in single myofibers, we measured the transcriptional contribution of satellite cells in our RNA preparation, since the isolation protocol cannot clear them completely from fibers. We compared the expression of Paired Box Homeotic Gene 7 (Pax7), CD56, Myogenic factor 5 (Myf5), Myogenic Differentiation 1 (Myod1) and Mrf4 in 8 single myofibers versus satellite cells. Pax7, CD56, Myf5 and Myod1 transcripts, that are known as markers of satellite cells (33,34), were found under-expressed in the RNA preparation of both fast and slow myofibers. On the contrary, Mrf4, that is highly expressed in adult skeletal muscle (35), was found less expressed in satellite cells (Supplementary Figure S2). Therefore, even if some satellite cells may actually remain attached to myofibers after their purification, their contribution to the fiber transcriptome can be considered absolutely negligible. It is known that miRNAs participate in the modulation of muscle stem cell behavior (36). However, even if the portion of RNAs from satellite cells had been more prominent than we actually measured, it would be considered fiber-specific and congruent with fiber specific phenotype (37). In fact, different works suggest that fiber type is dependent, at least in part, on the intrinsic properties of the progenitors which contributed to their generation (38,39). Then, we analyzed the expression of lncRNAs in 10 glycolytic type 2b and 11 oxidative type 1 myofibers characterized as previously described (18–20) (Supplementary Figure S3). This analysis allowed a more precise evaluation of the impact of lncRNAs in the specification of myofiber characteristics since it avoided interferences form non-contractile cells, such as endothelial, blood or connective cells. A total of 3242 microarray probes measured expression values statistically higher than background in at least 50% of tested type 1 or type 2b myofibers (Supplementary Table S3) with 264 probes that resulted preferentially expressed in a specific fiber type (Figure 1A and Supplementary Table S4). Interestingly, differentially expressed lncRNAs perfectly separated slow type 1 from fast type 2b myofibers indicating that they can be involved in processes activated or inhibited specifically in each myofiber type (Figure 1A). Most lncRNAs differentially expressed between glycolytic and oxidative myofibers are pseudogenes, ∼21% are long intergenic non-coding RNAs (lincRNAs) and ∼8% are antisense RNAs (Figure 1B). Microarray data confirmed that linc-Myh (2310065F04Rik), whose role in myofiber type specification is known (40), was highly expressed in fast 2b myofibers (Supplementary Table S4). To support microarray results we performed qPCR analysis for nine lncRNAs (3 showing higher expression in slow myofibers and 6 with the opposite expression profile). We included in this analysis functionally characterized lncRNAs, such as Gas5 (41), Neat1 (42), Dancr (43), Pvt1 (44–46) and 2310065F04Rik (linc-Myh) (40) and lncRNAs with no associated function. For this experiment, we used a new set of 8 fibers and for all lncRNAs the microarray data were confirmed. The lncRNA 1110020A21Rik did not evidence statistically significant difference in the expression between fast and slow myofibers but maintains the expression trend showed with microarray analysis (Figure 1E). Our results on the expression of lncRNAs of single myofibers could be useful for a better comprehension of different phenomena occurring in fast and slow muscles. For example, Gas5 could be associated to the different responsiveness of type 2 and type 1 myofibers to glucocorticoids (GC). In fact, abnormal levels of GAS5 may repress GC effectiveness (41). It is known that GC have exert a stronger metabolic impact in type 2 myofibers than in type 1 (47) where Gas5 is more expressed (Figure 1E). Some lncRNAs with a defined role in muscle biology, such as ^CE^RNA (48), linc-MD1 (49) and Munc (50) were not included in the microarray analysis because they were not present in the Ensemble 74 database, that we used for the definition of lncRNAs. For these lncRNAs, we performed qPCR analysis on four fast and four slow myofibers (Supplementary Figure S4) evidencing that linc-MD1 is more expressed in slow myofibers. This result confirms previous findings (49) where it was showed that linc-MD1 is only expressed in soleus muscle. ^CE^RNA and Munc, instead, did not show a fiber type specific expression.

### LncRNA localization in C2C12 cells and in single skeletal muscle fibers

LncRNAs can accomplish different functions depending on their subcellular localization. Nuclear lncRNAs are known to modulate gene expression through their interaction with chromatin (51) while cytoplasmic lncRNAs can function as miRNA sponges (49). We analyzed the subcellular localization of a group of lncRNAs in the C2C12 cell line—a recognized model to study skeletal muscle *in vitro*—using in parallel FISH and qPCR on RNAs extracted from nuclei and cytoplasm of the same fibers. The two methods gave similar results. According to qPCR results, after 14 days of differentiation the selected lncRNAs were prevalently localized in the nuclear fraction except for Gm16062, H19 and Igf2os that showed instead a cytoplasmic localization (Supplementary Figure S5). FISH analyses evidenced that Airn and Mir143hg, had a strictly nuclear localization while H19, Neat1, Nctc1 and Pvt1 evidenced a nuclear and cytoplasmic localization in elongated myoblasts—a characteristic phenotype acquired upon induction of differentiation (Supplementary Figure S6). The identification of Neat1 in the cytoplasm was already described in (6).

**Figure 2.**
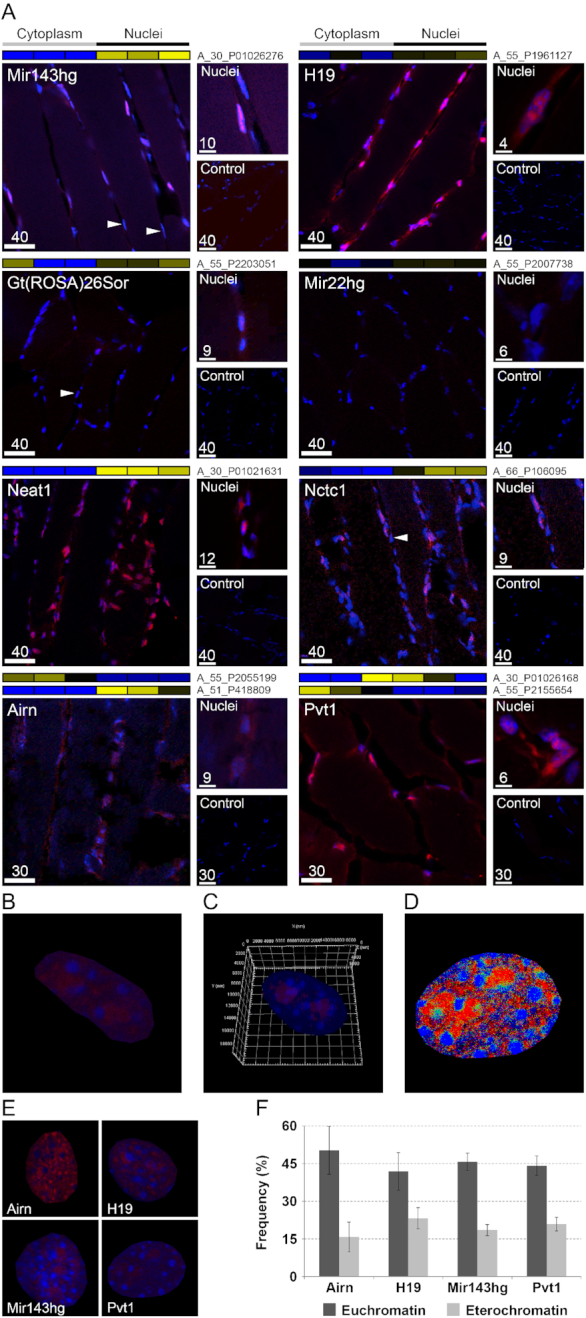
FISH of lncRNAs. (**A**) FISH on TA slices for selected lncRNAs. Labeled antisense strands of lncRNA probes were used as controls. Heat maps associated to each FISH images represent the results of genome wide analysis of lncRNA subcellular localization in TA myofibers. Names of microarray probes are listed near the heat map, where two names are present, it means that two different probes identified the same lncRNA in the microarray. Labels ‘Cytoplasm’ and ‘Nuclei’ indicate the cellular compartment where the signal of the probe was measured (blue = low expression; yellow = high expression). Arrows indicate examples of nuclei in the same myofiber that respond differently for the staining of some lncRNAs. Scale bar lengths are expressed in μm. (**B**) Enlarged FISH image of myonucleus positive for Pvt1 probe. (**C**) 3D reconstruction of a nucleus labeled for Pvt1 (red) and with DAPI (blue) (see also Supplemental Video S1). (**D**) Co-localization map. Pixels were colored in red where Pvt1 showed high expression, in blue when DAPI produced strong staining and in green when both had high staining. Co-localization (green) is underrepresented compared to individual Pvt1 or DAPI staining indicating that Pvt1 localizes in euchromatic regions. (**E**) Example of nuclei positively responding to probes for the lncRNAs Airn, H19, Mir143hg and Pvt1. (**F**) Frequency of high staining for lncRNA (red) in association with chromatin state (blue intensity). Chromatin state can be evidenced by DAPI staining. Dense areas of condensed chromatin (heterochromatin) correspond to DAPI-brighter regions. Airn, H19, Mir143hg and Pvt1 prevalently localized in euchromatic regions. Standard deviation was calculated on ∼20 nuclei per lncRNA.

To analyze lncRNA localization in myofibers, using a genome-wide approach, we applied a new method based on RNA purification from single subcellular components and microarray hybridization (Supplementary Figure S3). Here, we analyzed myofibers purified from EDL, *soleus* and TA mouse muscles identifying 481 lncRNAs predominantly localized in the myonuclei and 655 in the cytoplasm. Interestingly, 297 lncRNAs, that we named ‘Mixed’, showed a different localization depending on the specific isoform actually probed or on the type of muscle (Figure 1C, and Supplementary Table S5). Nuclear lncRNAs are prevalently pseudogenes (∼65%) while cytoplasmic or mixed lncRNAs are (lincRNAs ∼34%) or pseudogenes (∼31%) (Figure 1D). To validate these data, we performed FISH experiments on skeletal muscle sections for eight different lncRNAs (Figure 2A). We confirmed the nuclear localization for the lncRNA Mir143 hosting gene (Mir143hg), Gt(ROSA)26Sor, Neat1 and Nctc1. Mir22hg was chosen as an example of lncRNA with low expression to test the sensitivity of our FISH protocol. H19, Pvt1 and Airn showed a mixed localization: they were detected both inside the nucleus and in the cytoplasm. As already noticed in C2C12 cells (Supplementary Figure S6), it is of interest that the FISH of nuclear lncRNAs showed a patchy ‘clodded’ pattern suggesting a preferential localization and binding to specific chromatin regions (Figure 2B, C and D). Using 4′,6-diamidino-2-phenylindole (DAPI) staining, each nuclear region was categorized as eu- or hetero-chromatinic, evidencing that most of the lncRNAs are localized within euchromatic regions (Figure 2E and F). Moreover, we observed that not all nuclei in the same myofiber showed a similar pattern of lncRNA distribution (Figure 2A), suggesting a differential activity of myonuclei belonging to the same fiber as already evidenced for coding genes (52).

### Involvement of lncRNAs in skeletal muscle atrophy

Muscle atrophy is an important condition associated to several pathologies and correlates with patient’s vital clinical end points (53). In many cases muscle atrophy induces significant systemic metabolic modifications (54,55). To evaluate, if lncRNAs can be involved in this pathologic condition of the muscle, we tested the expression of 22 lncRNAs in different mouse models of atrophy. A few of the selected lncRNAs were already known for their involvement in muscle physiology (e.g. H19, Neat1). The function of the remaining lncRNAs is still unknown and they were selected because of their fiber type specificity. As models for muscle atrophy, we used denervation and ALS. In both atrophy models, mitochondrial fragmentation is a key feature, that can also be associated to apoptosis and autophagy processes. Seven coding RNAs were included in this analysis because their genomic location was adjacent to at least one of the chosen lncRNAs. We measured how the expression of these transcripts evolved during atrophy progression by testing lncRNA levels at three time-points for denervation (3, 7 or 14 days after surgery) and for ALS (1, 3 or 4 months of age).

**Figure 3.**
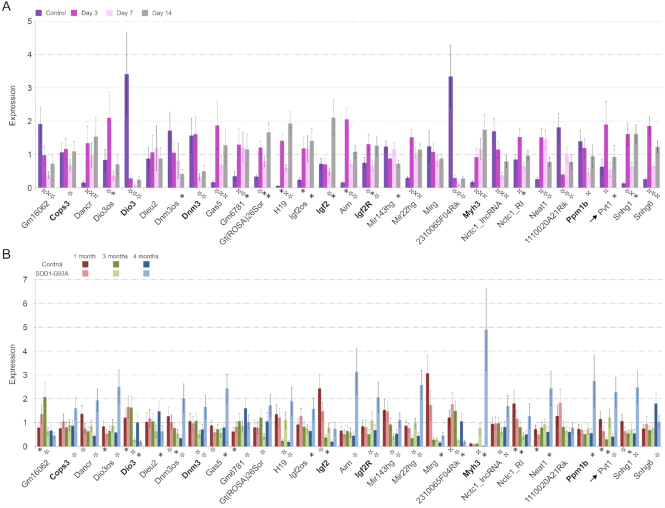
Expression of lncRNAs during muscle atrophy. Histograms represent expression values relative to the average expression of the gene among samples. Tbp was used as control gene. (**A**) Expression during denervation. Analyses were performed on *gastrocnemius* and a pool of RNAs extracted from controlateral non-denervated muscle was used as control. (**B**) Expression during ALS progression. As for denervated muscles *gastrocnemius* was used and transgenic mice with non-mutated human SOD1 gene were used as controls. Coding genes sharing the genomic location of the lncRNAs that are reported on their right side in the graph are indicated in bold. Arrows indicate Pvt1. Standard deviation among three biological and two technical replicates is indicated. The statistical significance between considered time points was computed using analysis of variance Student *t*-test for two-tailed distribution and unequal variance. * *P* ≤ 5 × 10^−2^, ☆ *P* ≤ 1 × 10^−2^, *P* ≤ 1 × 10^−3^.

Most lncRNAs were found differentially expressed compared to the control in at least one analysed condition. Generally, the expression of lncRNAs increased in muscles undergoing atrophy (Figure 3A and B). We found that 2310065F04Rik (linc-Myh) resulted down-regulated in both atrophy models while Dancr, Gas5, H19, Igf2os, Airn, MiR22hg, Neat1 and Snhg1 were up-regulated in the late phases of atrophy. As previously found, linc-Myh was associated to myofiber type specification. Its expression in nuclei of fast-type myofibers prevents slow-type and enhances fast-type gene expression (40). Our results support the importance of changes in myofiber type during muscle atrophy and show that a possible origin of the myofiber switch may be the down-regulation of linc-Myh. Most lncRNAs evidenced a time-dependent expression. During denervation, there was a peak of expression for most lncRNAs at day 3 after sciatic nerve resection, whereas in ALS mouse model this peak was detected at month 4 after birth. Since lncRNAs can influence the expression of adjacent genes (40), we evaluated whether the expression of each lncRNA and its adjacent coding RNA was coordinated. The expression of the couples H19-Igf2, Airn-Igf2r, 2310065F04Rik (linc-Myh)-Myh3 and Dnm3os-Dnm3 had a strong correlation in muscles of both models that, in fact, share common pathological features (56) (Supplementary Figure S7). Relationships among H19 and Igf2, and Airn and Igf2r were already known (57,58).

Interestingly, the expression of Pvt1, a lncRNA involved in cell cycle regulation (59), was increased during muscle atrophy (Figure 3). Pvt1 expression highly increased after 3 days from denervation, while during ALS progression Pvt1 expression was highly increased at 4 months of age when the pathology is reaching its terminal stage. This peculiar activation could be the result of the involvement of Pvt1 in processes of muscle atrophy such as, for example, energetic/metabolic changes (60), apoptosis (61) and autophagy (62). In fact, Pvt1 has been already associated to metabolic disorders such as diabetes (44,45).

### The role of lncRNA Pvt1 in skeletal muscle: *in vitro* and *in vivo* analyses upon silencing

**Figure 4.**
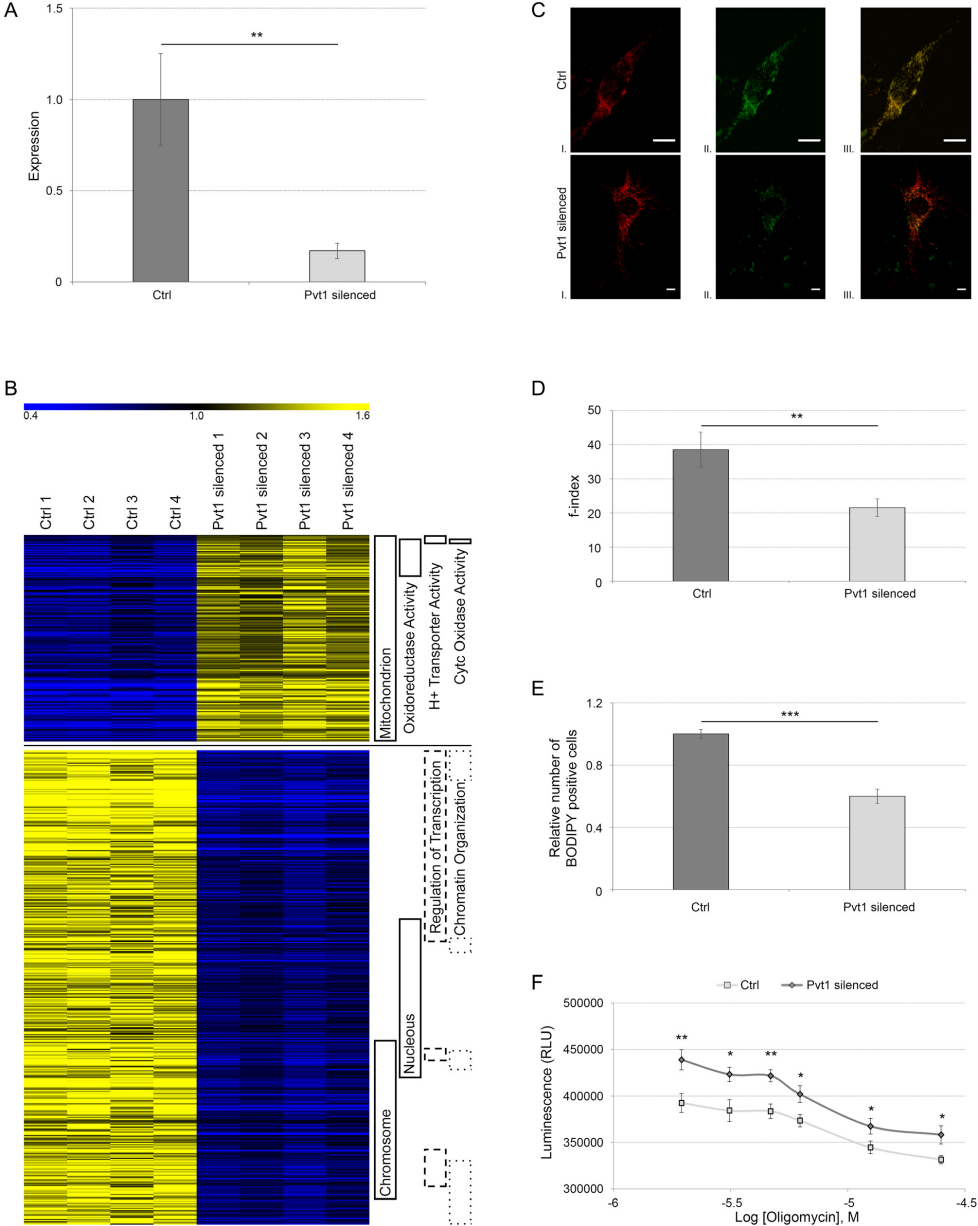
*In vitro* analysis of Pvt1 function. (**A**) Relative gene expression of Pvt1 in C2C12 cells transfected with control GapmeRs or with Pvt1 specific GapmeRs. Standard deviation is calculated among four biological replicates represented on the part B of this figure and two technical replicates per biological sample. Statistical significance was calculated according Student’s *t*-test between the two groups of cells with a two-tailed distribution and unequal variance; ** *P* ≤ 1 × 10^−2^. (**B**) Heat map of differentially expressed genes after Pvt1 silencing in C2C12 cells. On the right are indicated functional categories for genes represented. Up-regulated genes in Pvt1 silenced cells were involved in mitochondrial metabolism and respiration, while down-regulated genes were associated to transcription regulation. (**C**) Representative microscopy images of single C2C12 cells with different levels of mitochondrial fragmentation (I). Mitochondria were stained with Mito-RFP and imaged by confocal fluorescence microscopy. Images of each cell were captured at different focal depths and then processed. Cells were also stained with BODIPY (II) evidencing that after Pvt1 silencing BODIPY staining is less intense than control. Mito-RFP and BODIPY staining were merged (III). Scale bar represents 8 μm. (**D**) Quantization of mitochondrial fragmentation index. After Pvt1 silencing, mitochondrial fragmentation decreases indicating that mitochondria are more interconnected. Standard deviation was calculated from at least 50 different cells; ** *P* ≤ 1 × 10^−3^. (**E**) Relative cell count stained with BODIPY. A lower number of cells (50%) was stained with BODIPY when Pvt1 was down-regulated. Standard deviation was calculated from four different biological replicates; *** *P* ≤ 1 × 10^−4^. (**F**) ATP production in C2C12 cells treated with decreasing concentration of oligomycin. After Pvt1 silencing the ATP production was higher than in controls; * *P* ≤ 3 × 10^−2^, ** *P* ≤ 6 × 10^−3^. Statistical significance for f-index, BODIPY staining and ATP production was calculated using Student’s *t*-test between the two groups with a one tailed distribution and unequal variance.

To better characterize the function of Pvt1 in skeletal muscle, we tested the transcriptome of C2C12 muscle cells after Pvt1 down-regulation. We obtained ∼70% of Pvt1 silencing after treating cells with GapmeRs (Figure 4A). We evidenced that the down-expression of Pvt1 induced the down-expression of genes coding for nuclear proteins involved in the chromatin organization and gene transcription, and the up-regulation of genes coding for proteins involved in cell metabolism and mitochondrial function (Figure 4B and Supplementary Table S6).

#### Mitochondrial network and lipid consumption after Pvt1 silencing in C2C12 cells

We initially demonstrated that Pvt1 was preferentially expressed in (i) fast myofibers (Figure 1E), where mitochondria are fewer and less fused than slow myofibers and (ii) during muscle atrophy (Figure 3), when mitochondrial fragmentation increases. Moreover, we evidenced that the down-regulation of Pvt1 impacts the expression of mitochondrial related genes (Figure 4B). For these reasons, we tested if Pvt1 could have an effect on the mitochondrial network. We evidenced that, after Pvt1 silencing, mitochondrial network resulted less fragmented than controls (Figure 4C and D). Lipids are the main metabolic fuel in heart and skeletal muscle, and β-oxidation represents their main degradation pathway (63). Since we evidenced that after Pvt1 down-regulation mitochondria elongate and C2C12 cells decrease their lipid content (Figure 4C and E), we evaluated ATP production by promoting oxidative phosphorylation. We demonstrated that, replacing glucose with galactose in the culture medium, C2C12 cells exhibited higher ATP production when Pvt1 transcript was reduced (Figure 4F).

#### In vivo Pvt1 function

**Figure 5.**
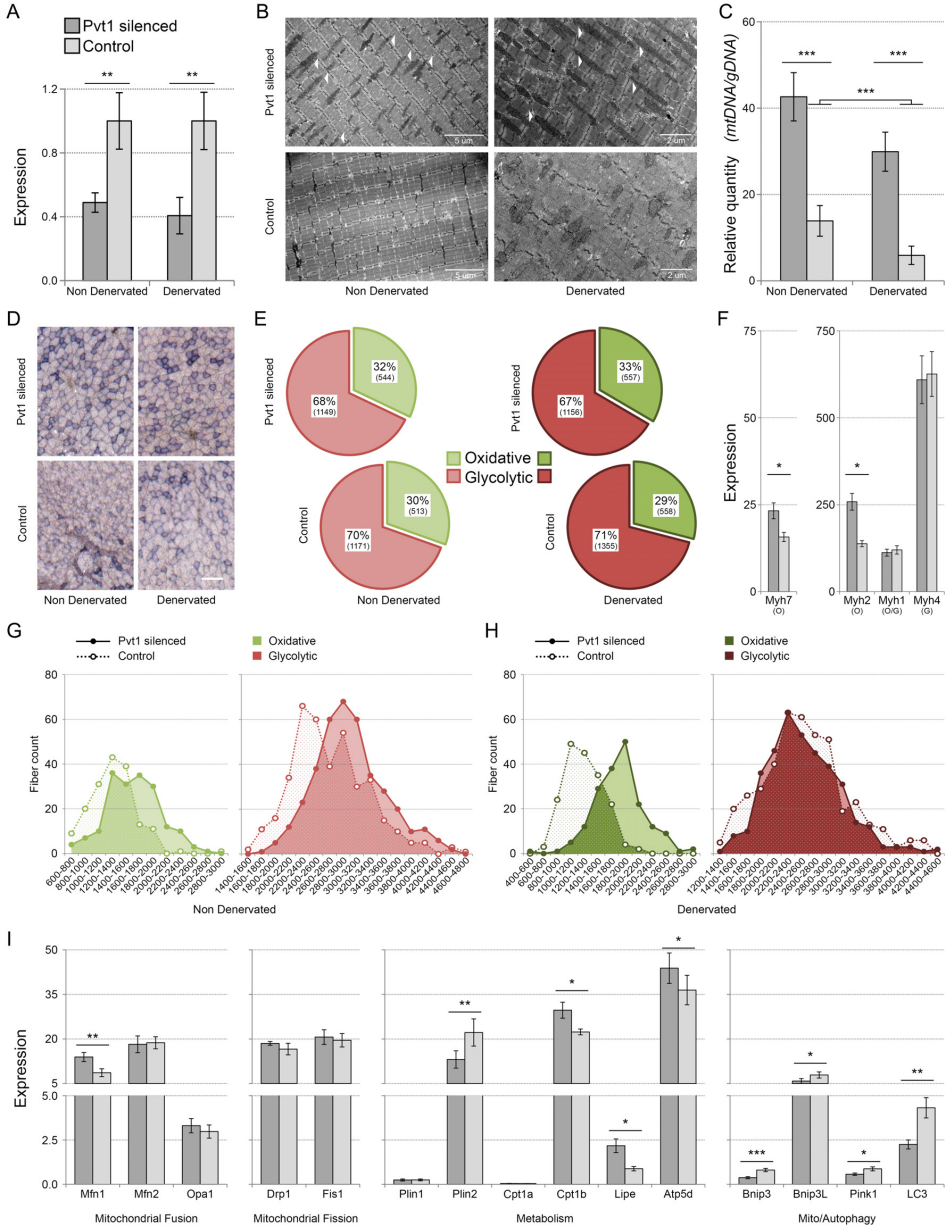
*In vivo* analysis of Pvt1 function. (**A**) Relative expression of Pvt1 after silencing with GapmeRs. Standard deviation was calculated among three biological and two technical replicates per biological sample. (**B**) Electron microscopy of TA slices. White arrows indicate elongated mitochondria in samples where Pvt1 was low (in both normal and denervated muscles). When Pvt1 was down-regulated, the number of mitochondria increased. (**C**) Quantization of mitochondrial mass. Mitochondrial mass was expressed as relative quantity of mitochondrial DNA coding for Cox compared with nuclear DNA coding for Sdh. It increased when Pvt1 was down-regulated. The genomic region coding for TBP was used as reference. Four biological replicates were analyzed. Standard error is represented. (**D**) Representative images for Sdh staining in *gastrocnemius* muscles. Scale bar represents 100 μm. (**E**) Percentage of oxidative and glycolytic myofibers is represented. χ² tests reveal that the increase in oxidative fibers after Pvt1 silencing is not significant for non-denervated muscles (χ^2^ = 1.09, *P*-value = 0.296) while it is significant for denervated muscles (χ^2^ = 4.75, *P*-value = 0.029). About 3500 myofibers for each condition were counted. (**F**) Histograms represent expression values relative to control gene (Tbp), obtained by qPCR for transcripts for myosin heavy chain. After Pvt1 silencing, myosin heavy chain genes expressed in oxidative metabolic myofibers (Myh2 and Myh7) increased their expression. On the contrary, genes expressed in glycolytic myofibers (Myh1 expressed in oxidative/glycolytic and Myh4 in glycolytic) were not affected. Standard deviation was calculated from three biological and two technical replicates per biological sample. O = Oxidative; G = Glycolytic (**G**) Cross-section area (CSA) was measured for ∼1200 fibers. Values are expressed in μm^2^. Fibers were divided in groups having similar area and their frequency was plotted. In non-denervated muscles the CSA of oxidative and glycolytic myofibers slightly increased when Pvt1 was down-regulated. (**H**) In denervated muscles the CSA of oxidative myofibers does not decrease when Pvt1 was down-regulated while it was unaffected for glycolytic myofibers. (**I**) Histograms represent expression values relative to control gene (Tbp) and normalized against the average expression of the gene among samples. Among tested genes involved in the mitochondrial dynamics only Mfn1 was up-regulated after Pvt1 silencing. Metabolic related genes showed instead the up-regulation of Cpt1b, Lipe and Atp5d and the down-regulation of Plin2. All genes associated to mito/autophagy were down-regulated in muscle where Pvt1 was down-regulated. Standard deviation was calculated among three biological and two technical replicates Statistical significance was calculated according to Student’s *t*-test between the two groups considering a two-tailed distribution and samples having unequal variance. * *P* ≤ 5 × 10^−2^, ** *P* ≤ 1 × 10^−2^, *** *P* ≤ 1 × 10^−3^.

To evaluate the role of Pvt1 *in vivo*, we inhibited the expression of this lincRNA in leg muscles of CD1 wild-type and denervated mice. We obtained a ∼50% down-regulation of Pvt1 in normal and ∼60% in denervated muscles (Figure 5A). The analysis of muscle ultrastructure by electron microscopy evidenced that mitochondria size and number increased after Pvt1 down-regulation (Figure 5B). We confirmed the increased mass of mitochondria by quantifying mitochondrial DNA content after Pvt1 down-regulation. Control denervated muscles evidenced a ∼50% reduction of mitochondrial mass compared to control non-denervated, while, after the Pvt1 silencing, mitochondrial mass increased both in denervated and non-denervated muscles. When Pvt1 was down-regulated, we evidenced only ∼30% of decrease in mitochondrial mass comparing normal and denervated muscles (Figure 5C). Myofibers in the *gastrocnemius* are known to be mostly fast; ∼54% are type 2b while type 2a and type 1 and 2x only account for 6 and 2% of the total fiber content (64). Using both succinate dehydrogenase staining and expression analysis of marker transcripts (Myosin Heavy Chain 7; Myh7, Myosin Heavy Chain 2; Myh2, Myosin Heavy Chain 1; Myh1 and Myosin Heavy Chain 4; Myh4), we showed an increase in the proportion of oxidative myofibers after Pvt1 down-expression (Figure 5D, E and F). This is in accordance with our previous findings of Pvt1 preferential expression in fast myofibers and in fast EDL muscle (Figure 1E and Supplementary Figure S8). When Pvt1 was down-expressed in normal muscles the expression of slow type 1 Myh7 was higher than in the controls. This indicates that the modulation of Pvt1 may alter myofiber type and metabolism; this aspect is further supported by the up-regulation of the Myh2 gene for fast oxidative myosin (Figure 5F). The Myh4 gene coding for the myosin heavy chain expressed in the type 2b fast glycolytic myofibers is not affected and, interestingly, neither the gene Myh1 that code for MyHC-2x expressed in type 2x fibers (Figure 5F). Type 2b and 2x fibers have a relatively low oxidative capacity compared to type 1 and 2a (9) despite the fact that they show moderate to strong SDH staining in rat skeletal muscle (65). In general, type 1 and 2a fibers primarily use oxidative metabolism, whereas type 2x and 2b fibers primarily rely upon glycolytic metabolism (66). Apparently, only the genes coding for myosin heavy chain proteins expressed in the two more oxidative myofibers (type 1 and 2a) were affected by Pvt1 silencing. Increases of SDH positive myofibers after Pvt1 silencing was statistically significant only in denervated muscles while in non-denervated muscles the increase was not statistically significant (Figure 5E). Since Pvt1 is up-regulated during muscle atrophy (in the first phases of muscle denervation), we tested if its down-regulation impacts the myofiber size in non-denervated and denervated muscles protecting them from atrophy induced by denervation. We evidenced that, in non-denervated muscle, cross section area (CSA) of both fast and slow myofibers slightly increased when Pvt1 is down-regulated (Figure 5G). A more interesting result was that in denervated muscles Pvt1 down-regulation was protecting from atrophy. In fact, when Pvt1 was down regulated, slow myofibers of denervated muscles had ∼40% higher CSA (1818 ± 350 μm^2^) compared to controls (1299 ± 273 μm^2^). Pvt1 silencing statistically impacted myofiber metabolism and dimension particularly during muscle atrophy induced by denervation supporting the functional importance of its up-regulation during this event. The fact that Pvt1 down-regulation protects from atrophy induced by denervation is evidenced by the ∼12.5% increase of slow myofibers mean CSA between non-denervated and denervated muscles. Fast myofibers were instead slightly affected by the down-regulation of Pvt1 (Figure 5G and H).

We evidenced that, after Pvt1 down-regulation in C2C12 cells, the mitochondrial network was altered (Figure 4C and D). We therefore checked the expression of genes coding for proteins involved in the modulation of the mitochondrial network after Pvt1 down-regulation in normal muscle, to avoid the effects induced by mechanisms activated during atrophy processes. These experiments showed that the expression of genes involved in mitochondrial dynamics did not change apart for mitofusin 1 (Mfn1) that was clearly up-regulated (Figure 5I). Since we previously evidenced higher levels of lipid metabolism after *in vitro* Pvt1 silencing, we further evaluated the expression of specific genes involved in lipid metabolism and ATP synthesis *in vivo*. We evidenced that the expression of genes involved in fatty acid catabolism such as Lipase E (Lipe) and Carnitine Palmitoyltransferase 1B (Cpt1b) increased while the gene Perlipin 2 (Plin2), a marker of lipid droplets, was down-regulated (Figure 5I). The up-regulation of Lipe, after *in vivo* down-regulation of Pvt1, is concordant with previous *in vitro* results. Since we evidenced that the down-regulation of Pvt1 causes an increase in ATP synthesis *in vitro*, we checked the expression of the ATP synthase gene (Atp5d) after Pvt1 silencing *in vivo* demonstrating that Atp5d was up-regulated (Figure 5I). All these results confirmed that the higher lipid consumption, probably associated with ATP production, previously found *in vitro*, also occurs *in vivo* after Pvt1 down-expression.

During muscle atrophy, mitochondrial fission is induced, triggering the remodeling of mitochondrial network through the autophagy system (67). Inhibition of mitochondrial fission also reduces muscle loss during fasting and after Forkhead box O3 (FoxO3) overexpression (67). Since we evidenced that mitochondrial network resulted less fragmented after Pvt1 down-regulation, we analyzed the expression of genes coding for proteins involved in mito/autophagy. We evidenced that all selected genes were down-regulated after Pvt1 down-regulation (Figure 5I) supporting the idea that myofiber mass loss was attenuated and mitochondria were protected from degradation, as already known for elongated mitochondria (68).

### Pvt1 limits autophagy and apoptosis through c-Myc

**Figure 6.**
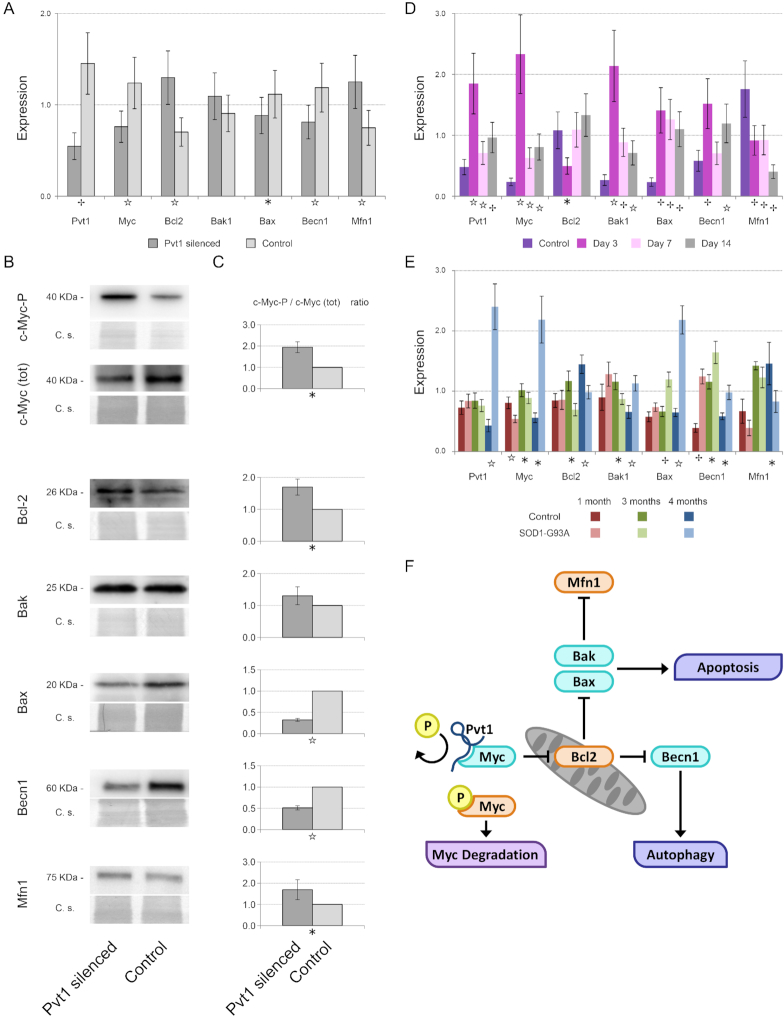
Mechanism of action of Pvt1 to prevent muscle atrophy. (**A**) Histograms represent expression value relative to the average expression of the gene among samples. Tbp was used as reference gene. After Pvt1 down-regulation, c-Myc, Bax, Beclin 1 were under-expressed while Bcl-2 and Mfn1 were over-expressed. (**B**) Western blots performed on target proteins of C2C12 cells where Pvt1 was silenced. Both immunoreactive bands (upper) and the corresponding Coomassie blue staining (C.s., lower) are shown (**C**) Densitometric analysis of western blots in which the optical density of immunoreactive bands was normalized to the optical density of the corresponding Coomassie-stained lane. Reported data are mean ± SEM, calculated among at least four biological replicates, and the statistical significance was assessed using one-tailed Student’s *t*-test between paired samples. (**D**) Histograms represent expression values relative to the average expression of the gene among samples. *Gastrocnemius* was used as muscle and Tbp as reference gene. During Denervation Pvt1 increases its expression at day 3 like c-Myc, Bak1, Bax and Beclin 1. On the contrary, Bcl-2 and Mfn1 were under-expressed. (**E**) Histograms represent expression value relative to the average expression of the gene among samples. *Gastrocnemius* was used as muscle and Tbp as reference gene. During ALS progression, Pvt1 expression is increased in 4-month-old ALS mice. c-Myc, Bak1, Bax and Beclin 1 expression concomitantly increases. On the contrary, Bcl-2 and Mfn1 were under-expressed. Statistical significance of qPCR experiments was calculated using a Student’s *t*-test between control and the considered time point or treatment for Pvt1 down-regulation with a two-tailed distribution and unequal variance. Standard deviation of qPCR was calculated among three biological and two technical replicates. (**F**) Cartoon representing the mechanism of action of Pvt1 during muscle atrophy. During muscle atrophy Pvt1 is up-regulated blocking c-Myc phosphorylation and degradation. In turn, Bcl-2 results up-regulated impinging on autophagy and apoptosis through the regulation of Beclin 1 and Bax. For the entire figure * *P* ≤ 5 × 10^−2^, ☆ *P* ≤ 1 × 10^−2^, *P* ≤ 1 × 10^−3^.

It is known that in cancer cells Pvt1 is able to prevent c-Myc degradation by hampering its phosphorylation on Thr58 (69). c-Myc is a known modulator of the anti-apoptotic protein Bcl-2 (70) that represents a central node in the regulation of both apoptosis and autophagy, two important events occurring during muscle atrophy. Bcl-2 is able to interact with Beclin 1, that in turn recruits key autophagic proteins to a pre-autophagosomal structure, preventing the formation of, and thus blocking, the autophagosomal structure and blocking it (71,72). On the other hand, Bcl-2 regulates the activity of the BCL2 Associated X/ BCL2 Antagonist (Bax/Bak) complex (73). The down-regulation of Pvt1 in C2C12 cells resulted in the altered expression of c-Myc, Bcl-2, Bax, Beclin 1 and Mfn1 as measured by qPCR (Figure 6A). These results also support microarray data (Supplementary Figure S9). These results were confirmed by western blot analyses of the correspondent proteins (Figure 6B). We evidenced that the ratio of phosphorylated c-Myc-P in the Thr58 over total c-Myc (Figure 6C) was determined by both an increase of c-Myc-P and a decrease of the total c-Myc expression. This result demonstrated that also in skeletal muscle cells Pvt1 interacts with c-Myc. Bcl-2 up-regulation occurring after Pvt1 down-expression correlated, as expected, with the reduced expression of Bax and Beclin 1, while, on the contrary, the expression of Bak was unaffected (Figure 6A, B and C). Apoptosis is strongly linked to mitochondrial conformation. Death-promoting Bcl-2 family members, such as Bax, can promote cytochrome c release and fragmentation of the mitochondrial network, whereas apoptosis-inhibitory members, such as Bcl-2 and Bcl-xL, antagonize these events. Two recent studies have reported that the anti-apoptotic Bcl-2 and Bcl-xL, and the pro-apoptotic Bax and Bak of the Bcl-2 family regulate mitochondrial morphology (15,16). We confirmed that mRNA and protein content of Mfn1, one of the most important regulators of mitochondrial fusion, were increased upon modulating Pvt1 (Figure 6A, B and C).

In summary, our findings suggest that Pvt1 is able to modulate the expression of c-Myc, which, in turn, regulates Bcl-2, ultimately impinging on autophagy and apoptosis that are strictly related to mitochondrial conformation. We confirmed this mechanism also in atrophy models that show Pvt1 up-regulation. After 3 days from denervation and in 4-month-old SOD1(G93A) transgenic mice, time points with the maximal expression of Pvt1, the level of mRNA for c-Myc was higher in comparison with controls, and the same was observed for Beclin 1, Bax and Bak (Figure 6D and E). Atrophy and fibrosis are strictly associated in skeletal muscle. In fact, the master regulator of fibrosis, transforming growth factor beta 1 (Tgfb1) (74), is able to trigger muscle atrophy as well (75). Moreover, in (76) it was evidenced that Pvt1 contributes to the deposition of extracellular matrix in the glomeruli. We analyzed the expression of genes associated with extracellular matrix deposition after Pvt1 modulation and we found that there is a strong correlation between their expression and Pvt1 expression (Supplementary Figure S10).

## DISCUSSION

LncRNAs are involved in different aspects of cell biology, such as the regulation of cell cycle (77), tumorigenesis (78), development (79,80), muscle differentiation and disease (81,82) and metabolism (83). One of the first lncRNA functionally characterized in skeletal muscle was lincRNA-MD1, a regulator of miR-133 and -135 (49). Recently, it was evidenced that miRNAs and lncRNAs expressed specifically by fast or slow contracting muscles contribute to the establishment of fiber-specific transcriptome (84). Although these results have demonstrated the importance of lncRNAs in muscle transcriptome specification, they are biased by the use of whole muscle samples that are heterogeneous in terms of cell type and myofiber composition. It is becoming clear that single cell studies provide a more focused tool to investigate transcriptional dynamics. Thus far, there is limited genome wide information about lncRNA expression in single cells (5) and no data exist regarding the myofiber specificity of these non-coding molecules. To lay the groundwork for the study of lncRNAs in skeletal muscle during development and disease, we generated a reference catalog of lncRNAs expressed at single myofiber level. This is useful to understand which lncRNA, included in Ensembl 74 database, is specifically expressed by each smallest contractile unit of skeletal muscle (the myofiber) without interference from other cell types that can be found in the whole muscle such as endothelial, blood cells or fibroblasts. Our analyses reveal that several lncRNAs are fiber specific, supporting the idea that lncRNAs can be involved in the regulation of myofiber specificity.

The subcellular localization is an important determinant of lncRNA functions. Therefore, it is fundamental to determine if they are prevalently localized in the nucleus or in the cytoplasm, the two main compartments where lncRNAs exert their functions. To map the lncRNAs in purified myofibers we used a new method that was originally developed by us to localize lncRNAs in ovarian cancer cells (78). We showed that the same lncRNA can occupy different subcellular compartments in different myofiber types. Interestingly, FISH experiments demonstrated that not all myonuclei belonging to the same fiber show a positive signal for a specific lncRNA, supporting the idea that nuclei may be functionally different depending on their location along the myofiber (85). We also evidenced that myonuclei in a single myofiber present a different pattern of chromatin-lncRNA binding. Moreover, the same subcellular localization of lncRNAs in C2C12 cells and in myofibers are comparable, indicating that this cell line is a suitable model for functional studies of lncRNAs. The results presented in this paper were confirmed by different techniques and also support published data such as the cytoplasmic localization of H19 lncRNA (6) or the expression of specific lincRNA in fast (40) or in slow (49) muscles.

Skeletal muscle is an extremely plastic tissue that responds to external stimuli by adapting its anatomy and physiology. In fact, muscle fiber composition can change with physiopathological modifications such as exercise or muscle atrophy. Muscle atrophy can affect specific fiber types, affecting predominantly slow type 1 or fast type 2 myofibers, frequently causing slow-to-fast or fast-to-slow fiber type shift. Motor neuron degeneration and muscle denervation cause a shift from slow to fast muscle phenotype (86–88) and an increase of mitochondrial fragmentation (89,90). Mitochondrial dynamics play a key role in muscle atrophy, in fact, mitochondrial fragmentation enhances muscle wasting while pharmacological inhibition of mitochondrial fragmentation is sufficient to dampen autophagy, as well as muscle atrophy (89).

We evidenced that lncRNAs expressed in muscle change their expression also during pathological conditions that induce muscle atrophy. Interestingly, Pvt1, a lncRNA that is conserved between mouse, rat and man (91) was up-regulated during muscle atrophy. Pvt1 has been extensively studied in cancer (92) where it is known to regulate the expression of c-Myc (46). Pvt1 was already linked to metabolism since its expression was found altered in autophagic processes of diabetic mice (93) and its expression is modulated by glucose (83). All these processes modulated by Pvt1 are important for the proper functioning of muscles in different situations: from cell differentiation where the expression of c-Myc is inversely related to the ability of forming myofibers (94,95), to atrophy where muscle mass loss is associated with the activation of autophagy (96), apoptosis (97) and metabolic changes (98). Our *in vitro* and *in vivo* analyses evidenced the influence of Pvt1 on mitochondrial morphology, that impacts the production of ATP by using lipids as energy substrate. In fact, the down-regulation of Pvt1 causes the up-regulation of genes coding for proteins involved in the hydrolysis of stored triglycerides such as Lipe, and in the initiation of the mitochondrial oxidation of long-chain fatty acids such as Cpt1a. At the same time, we highlighted the down-regulation of genes associated to lipid storage such as Plin2, the only constitutive and ubiquitously expressed lipid droplet protein. This ability strongly correlates with the enhanced expression of slow type myosin heavy chain coding genes, supporting the fact that mitochondrial dynamics influence muscle plasticity (14). We demonstrated that, when Pvt1 was down-expressed, muscles developed a resistance to atrophic processes after denervation. The mechanism is reliant on the ability of Pvt1 to modulate apoptosis and autophagy, two important pathways activated during muscle atrophy. After Pvt1 down-expression, c-Myc is destabilized and consequently the anti-apoptotic protein Bcl-2, a central node in the regulation of both apoptosis and atrophy, is up-regulated. Bcl-2 regulates the pro-apoptotic protein Bax, the activation of which, in mammalian cells, is preceded by mitochondrial fragmentation (99,100). In skeletal muscle, the down-regulation of Pvt1 impinges both Bcl-2 and Bax and increased the expression of Mfn1, an important protein for mitochondrial fusion (101). We also associated this result with a diminished expression of genes and proteins involved in mito/autophagy (Figure 6F). It is important to stress the fact that a fine equilibrium of mitochondrial fusion/fission processes is needed to preserve muscle mass and prevent muscle wasting.

In summary, our study demonstrates the importance of lncRNAs as regulators of skeletal muscle as a metabolically active tissue. This aspect would be of great interest for non-coding RNA-based diagnostic and therapeutic applications as shown also by our *in vivo* silencing experiments. In fact, non-coding RNAs are likely to represent viable therapeutic targets, or useful readouts of treatment efficacy or disease progression. This study lays the groundwork for future studies aimed at better understanding the fine-tuning of lncRNAs during muscle development, differentiation, growth, plasticity and disease.

## DATA AVAILABILITY

All genomic data were submitted to Gene Expression Omnibus database (GEO). Single myofiber lncRNA expression analysis: GSE112716; nuclear-cytoplasmic lncRNA localization: GSE112768; Pvt1 silencing: GSE112881.

## Supplementary Material

Supplementary Data
