# Supplementary material for: Single cell analysis reveals the involvement of the long non-coding RNA Pvt1 in the modulation of muscle atrophy and mitochondrial network
Source: Nucleic Acids Res. 2019 Jan 16;47(4):1653–70. doi: 10.1093/nar/gkz007 (PMC6393313; doi:10.1093/nar/gkz007)
Supplement: Supplementary Data [file gkz007_supplemental_files.zip › Supplemental Figures-ok-REVISION-DEF.docx]

**Figure S1. Myofiber lysis and nuclei purification. A.** After myofiber lysis, nuclei were stained with SYBR Safe and observed under the microscope to evaluate if they were free from cells. In the figure representative images of the over imposition of fluorescent and bright field images of nuclei purified from EDL myofibers. Nuclei were not included in any myofiber; in fact, they were scattered and not aligned. **B.** Comparison of cytoplasmic (blue) and nuclear (red) total RNA fractions by 2100 Bioanalyzer chip. Nuclear RNA profiles always had three unique peaks (arrows) not observed in the cytoplasmic RNA profile. This indicates no cross-contamination between the nuclear and cytoplasmic fractions as reported in (1).


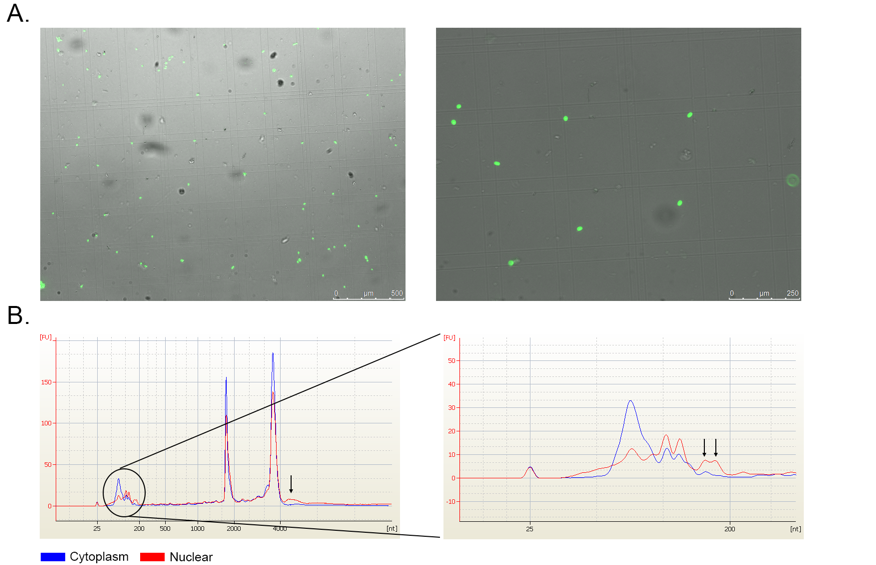


**Figure S2. Expression of markers of satellite cells.** qPCR was used to evaluate the contribution of satellite cells in our RNA preparations obtained from single myofibers. We analyzed 4 slow (green) and 4 fast myofibers (red) using satellite cells as controls (blue). Markers of ~~active~~ satellite cells (Pax7, CD56, Myf5, and Myod1) have considerably lower expression levels in myofibers compared to satellite cells. The expression of these genes is also much lower in our preparations compared to the expression of Mrf4, a gene highly expressed in adult skeletal muscle. Standard deviation represents the error of three technical replicates. Txn1 was used as reference gene. All statistical significances were calculated using Student’s t-test between myofibers and the control with a two tailed distribution and unequal variance. ** p ≤ 1 x 10^-2^, *** p ≤ 1 x 10^-3^.

**
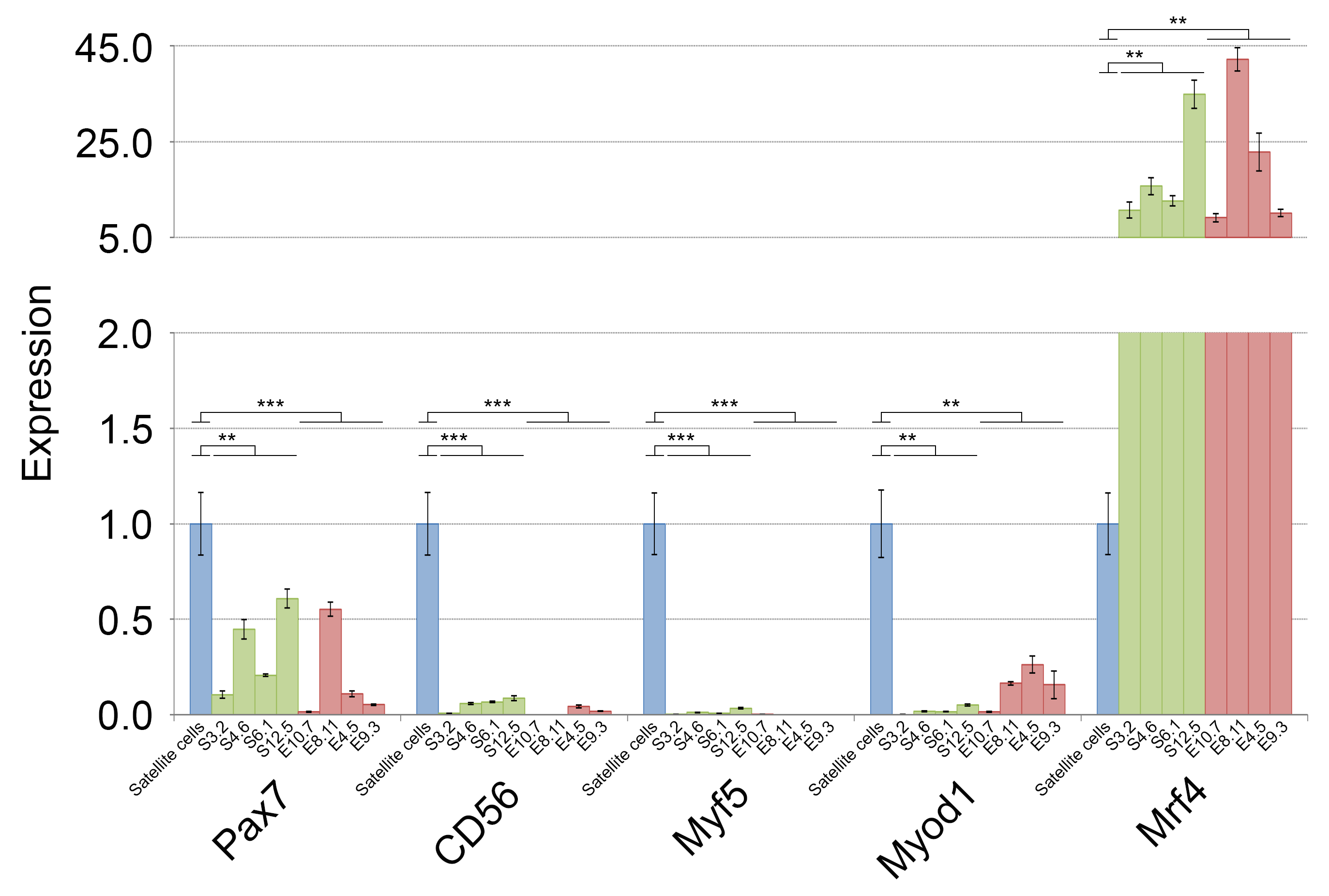
**

**Figure S3. Scheme of experimental design for the analysis of the expression of lncRNAs in single myofibers and for the identification of their subcellular localization.** Scheme reassuming the steps followed for the preparation of RNA samples used for the analysis of fiber type specificity and subcellular localization of lncRNAs. Myofibers were isolated from three different muscles: *extensor digitorum longus* (EDL, red), *tibialis anterior* (TA, yellow), and *soleus* (green). Pools of myofibers from the same muscle were used for the localization studies while single myofibers were used for expression profiling (fast = red; slow = green).


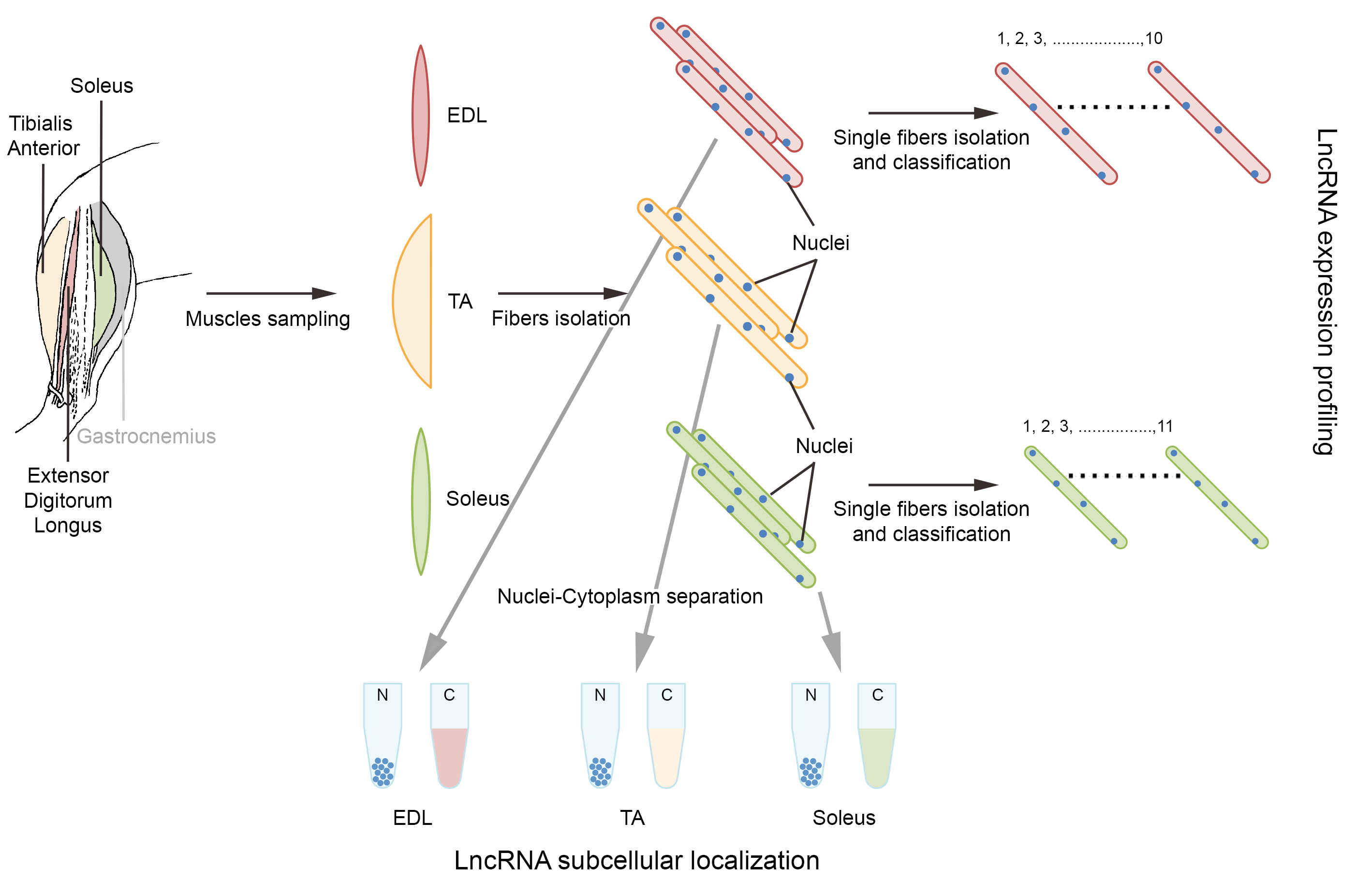


**Figure S4. Expression of ^CE^RNA, linc-MD1 and Munc in single myofibers.** qPCR was used to evaluate the expression in single myofibers of three lncRNAs known to be involved in muscle biology but missing in Ensembl 74. We analyzed 4 slow myofibers (green) and 4 fast myofibers (red). Standard deviation represents the error of three technical replicates. Linc-MD1 resulted over-expressed in slow myofibers compared to fast myofibers. Txn1 was used as reference gene. All statistical significances were calculated using Student’s t-test between groups of myofibers with a two tailed distribution and unequal variance. * p ≤ 5 x 10^-2^.

**
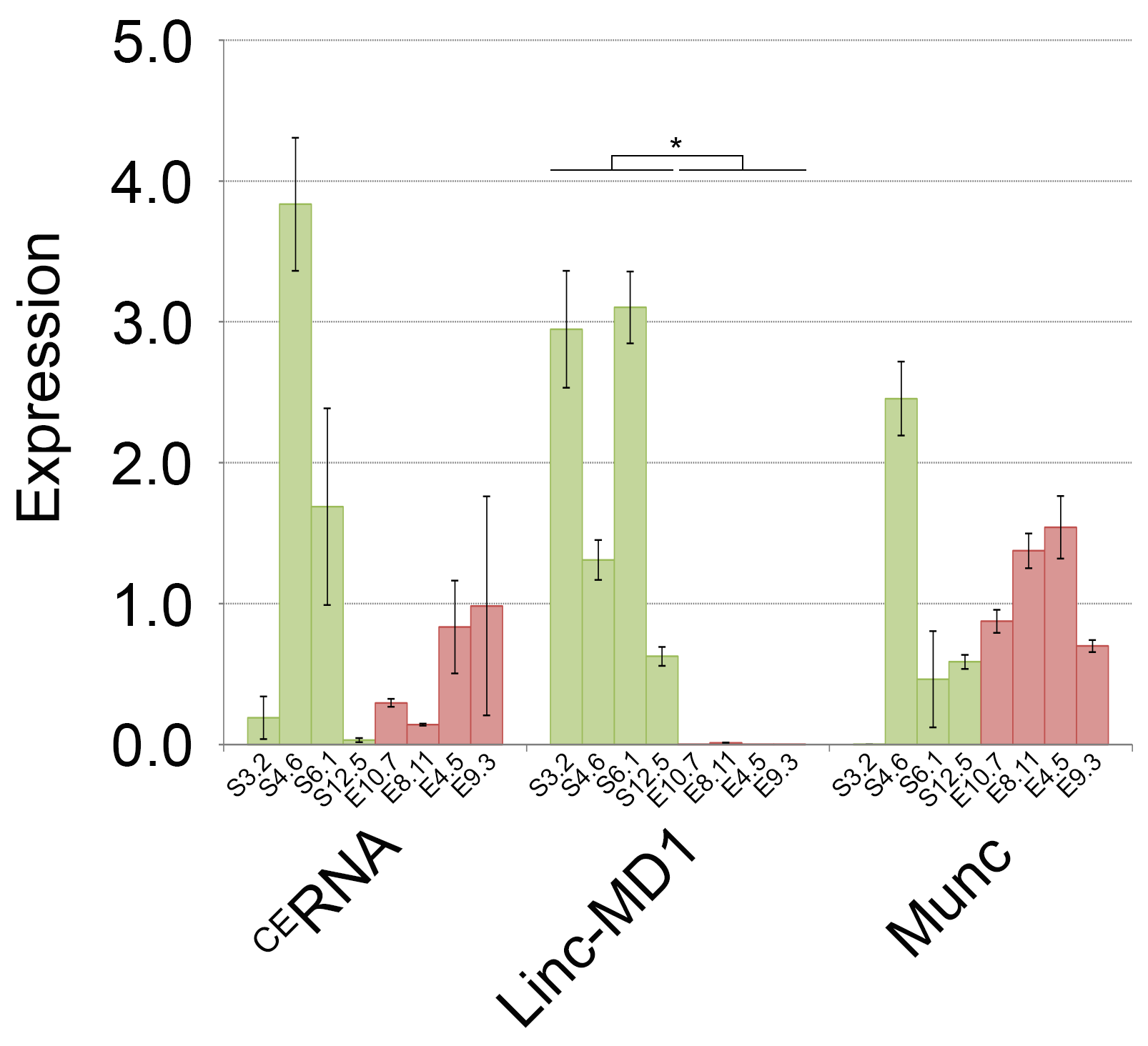
**

**Figure S5. Subcellular localization of lncRNAs during C2C12 differentiation.** qPCR was used to evaluate nuclear or cytoplasmic preferential expression of a group of lncRNAs during C2C12 differentiation. Chosen points were proliferating myoblasts and differentiating myoblasts at 1, 3, 7, or 14 days after adding differentiative medium. In red is represented cytoplasmic expression from proliferating myoblasts (light red) to 14 days of differentiation (dark red). In blue is represented the expression of the lncRNAs in the nuclear fraction (light blue proliferating medium; dark blue differentiating at 14 days). RI in the name of Nctc1 stays for retained intron isoform. Standard deviation represents the error of three biological and two technical replicates. Tbp was used as reference gene. Statistical significance was calculated using Student’s t-test between cytoplasm and nucleus at 14 days of differentiation with a two tailed distribution and unequal variance. * p ≤ 5 x 10^-2^, ** p ≤ 1 x 10^-2^, *** p ≤ 1 x 10^-3^.

**
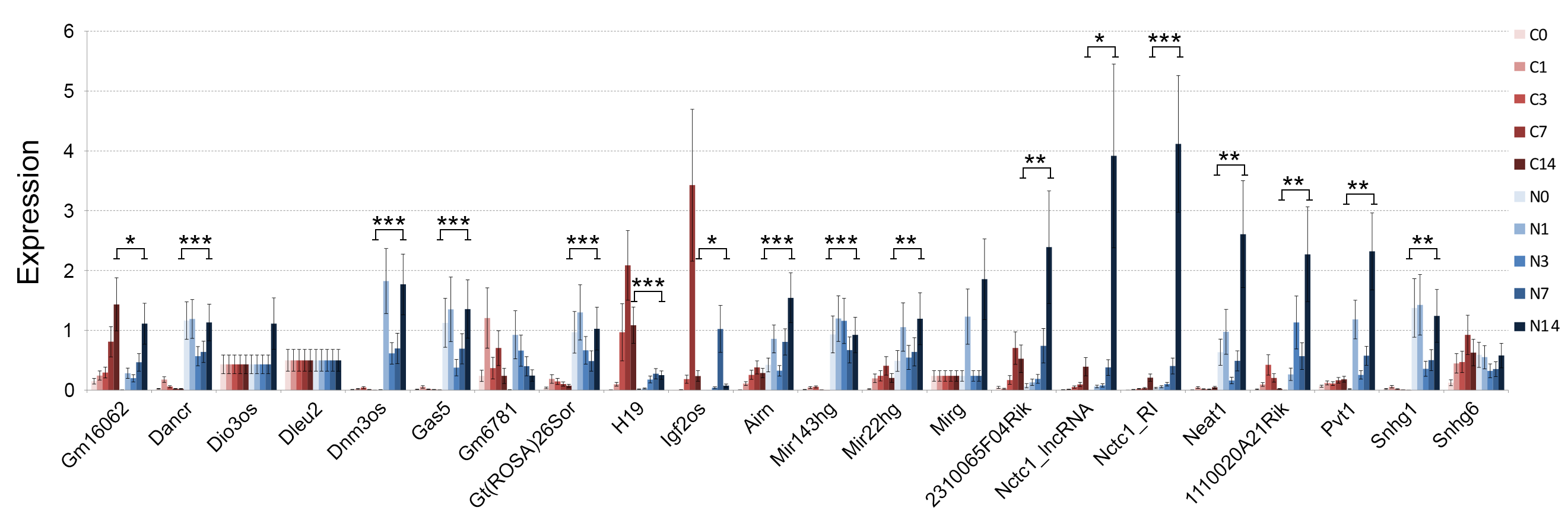
**

**Figure S6. FISH staining on C2C12 cells.** Representative images of FISH staining for different lncRNAs. Negative control was performed using a probe with the same sequence of the target to evaluate a-specific hybridization and to verify the absence of signal from the genomic sequence. Above each scale bar is indicated the dimension in µm. Arrows indicate some elongated cells for which the lncRNA is highly expressed in the cytoplasm.

**
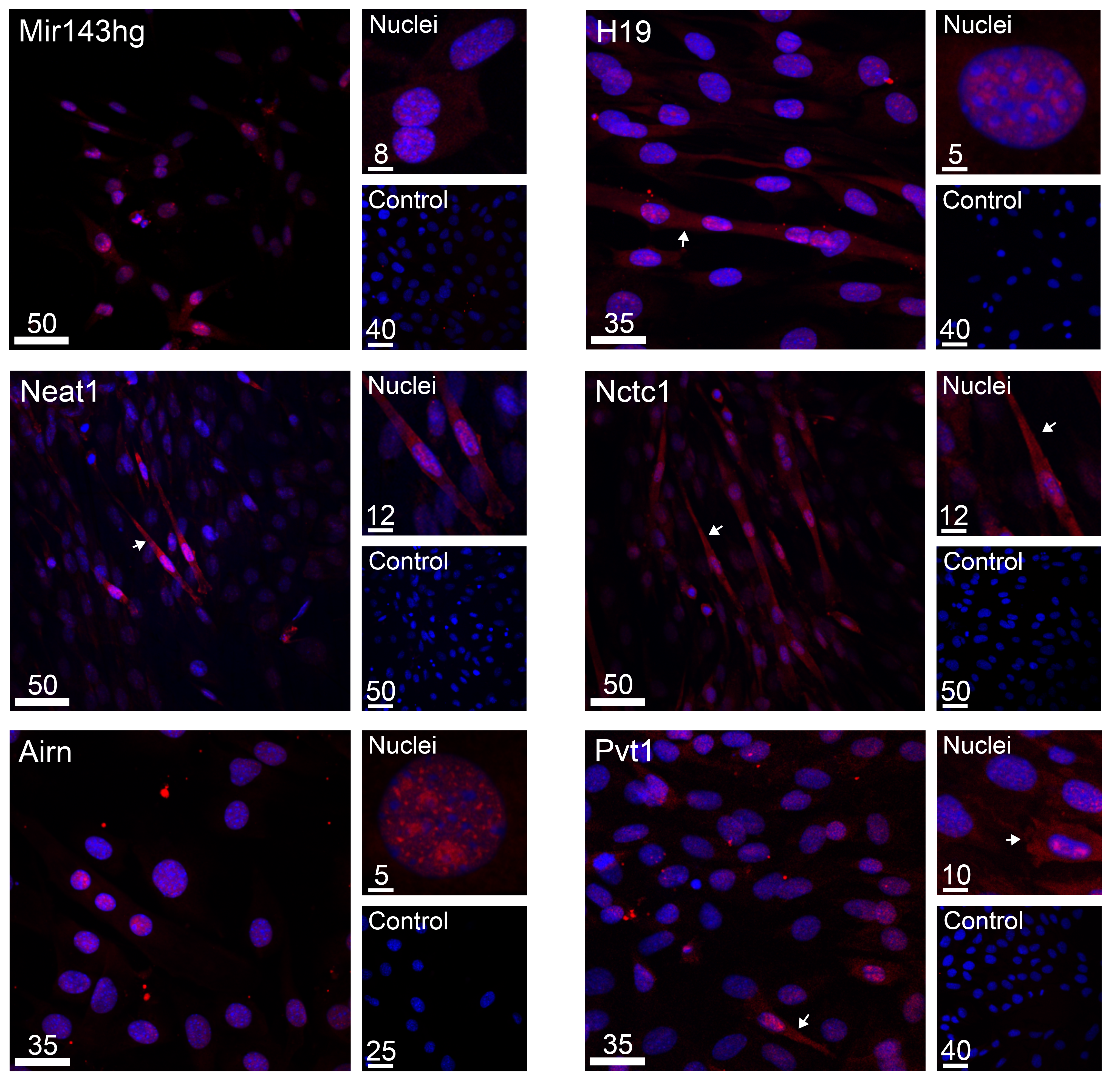
**

**Figure S7. Correlation between the expression of lncRNA and the adjacent coding gene.** Pearson correlation was used to identify gene expression inter-relationship between lncRNAs and adjacent coding RNA. Two isoforms of Nctc1 were tested: Nctc1_lncRNA and Nctc1_RI (Retained Intron). A pool of RNA extracted from non-denervated contralateral muscles was used as control for denervated muscles, while muscles from transgenic mice expressing the human wild-type SOD1 gene were used as controls for the transgenic mice expressing human mutated SOD1 gene (ALS model) always comparing mice with the same age.

**ALS model**

**Denervation**

**ALS model**

**Denervation**


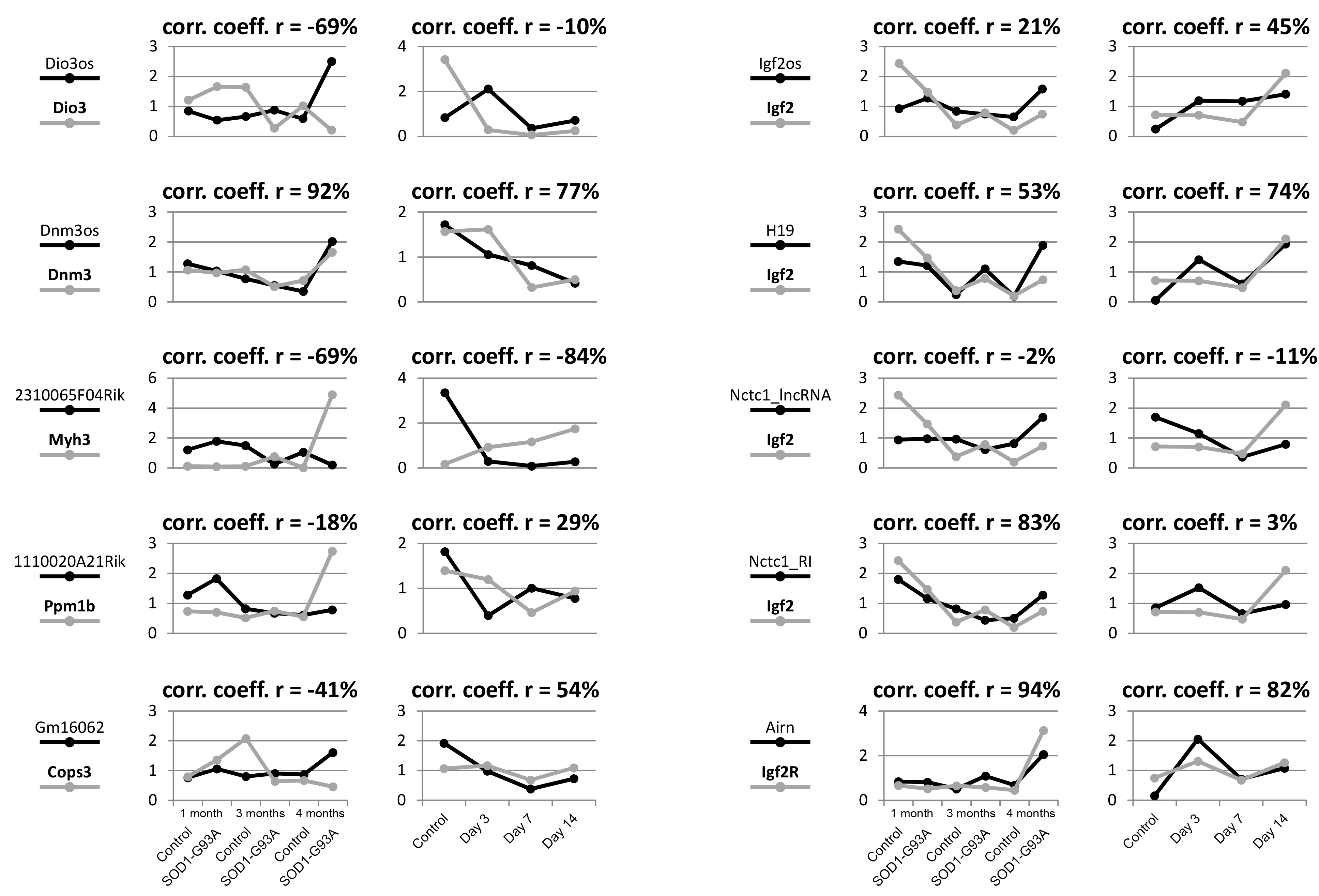


**Figure S8. Relative expression of Pvt1 in whole muscle.** qPCR was performed extracting RNA from whole muscles (EDL and Soleus) of normal mice. Standard deviation is for three biological and two technical replicates. Txn1 was used as reference gene. Statistical significance was calculated using Student’s t-test between the two conditions with a two tailed distribution and unequal variance. ** p ≤ 1 x 10^-2^.


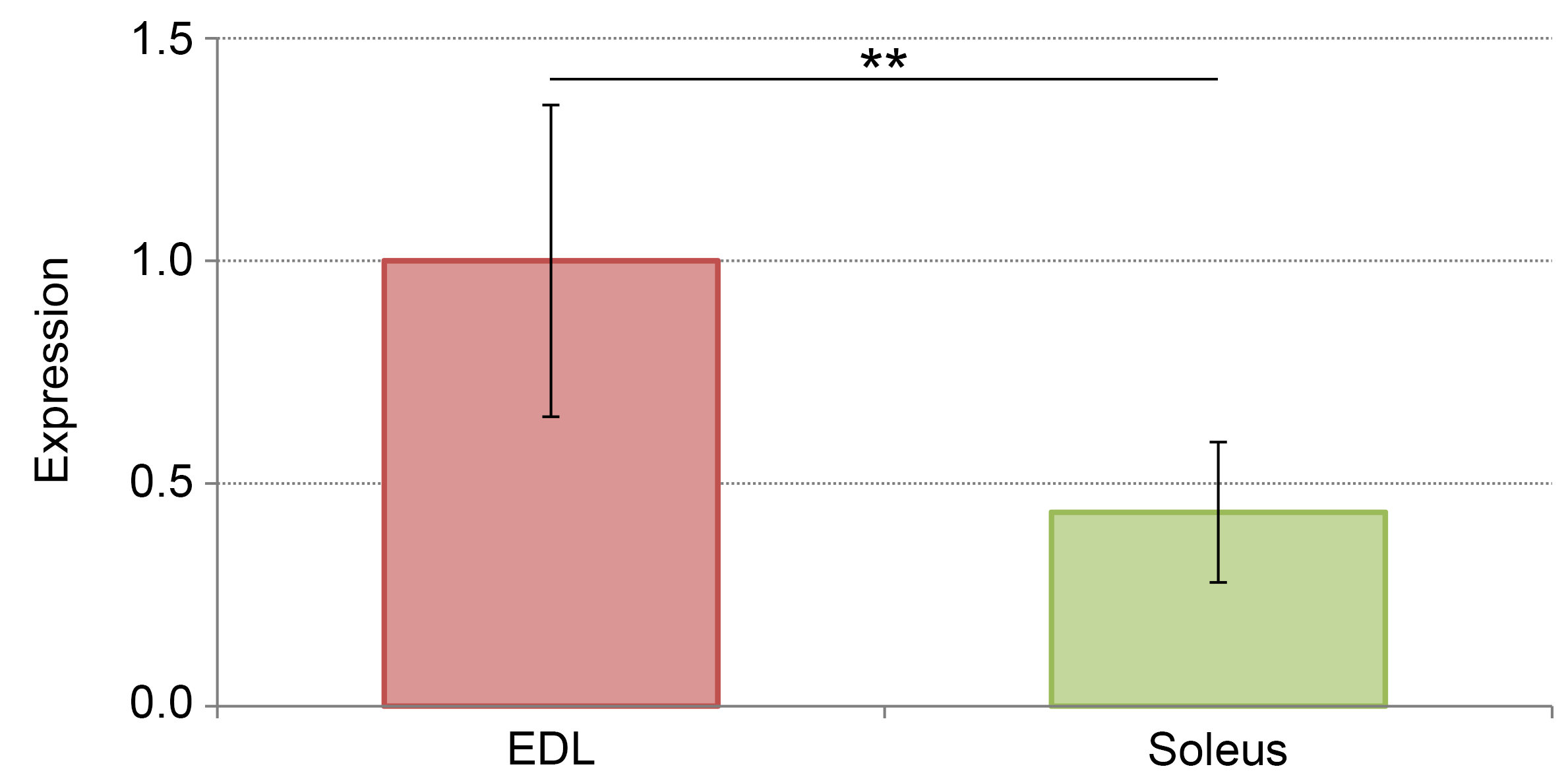


**Figure S9. Microarray gene expression.** Microarray analyses were performed to evaluate the effect of Pvt1 down-regulation on the expression of coding genes in C2C12 myoblasts. Pvt1 down-regulation was obtained treating C2C12 myoblasts with Pvt1 specific GapmeRs (dark grey) while controls were treated with GapmeR Negative Controls provided by the manufacturer (light grey). Standard deviation is calculated among four biological replicates. Statistical significance was calculated using Student’s t-test between the two conditions with a two tailed distribution and unequal variance. * p ≤ 5 x 10^-2^, ** p ≤ 1 x 10^-2^, *** p ≤ 1 x 10^-3^.


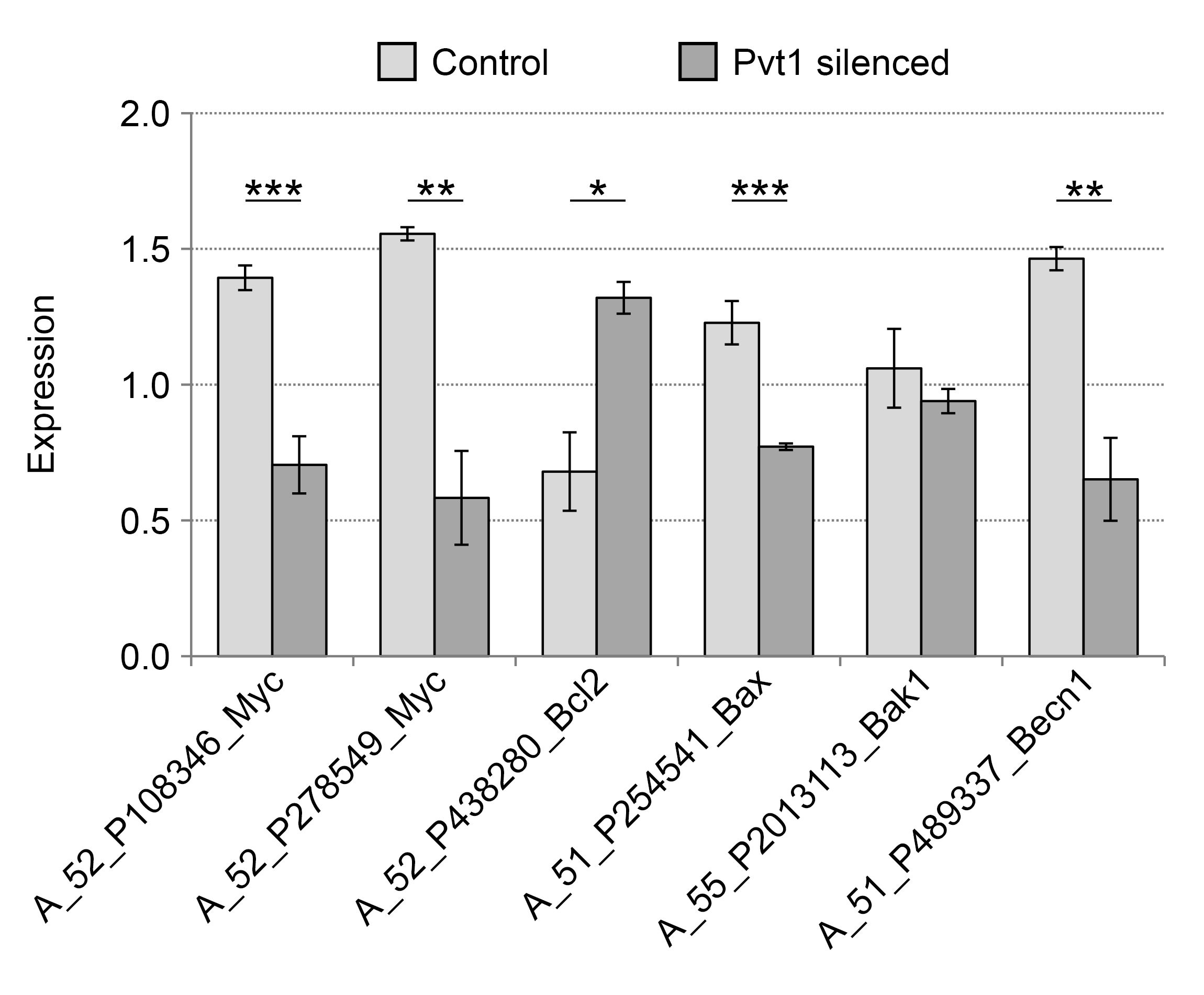


**Figure S10. Expression of genes involved in the fibrosis in association with the expression of Pvt1.** qPCR was performed using RNA from different biological samples to evaluate the expression of protein coding genes known to be involved in the fibrosis. **A.** Analysis of C2C12 myoblasts transfected with Pvt1 specific GapmeRs (dark grey) to down-express Pvt1 and Negative Controls GapmeR provided by the manufacturer (light grey). The down-expression of the master regulator of fibrosis, Tgfb1, and other fibrosis related genes, corresponds to the down-regulation of Pvt1. Standard deviation is for three biological and two technical replicates. B2m was used as reference gene. **B.** Gene expression in denervated muscles compared with contralateral non-denervated muscles. Muscles were collected 3, 7 or 14 days after denervation. Standard deviation is for three biological and two technical replicates. Tbp was used as reference gene. **C.** Gene expression in muscles derived from ALS mouse models. Standard deviation is for three biological and two technical replicates. Tbp was used as reference gene. In both denervated and ALS models the up-regulation of Pvt1 corresponds to the up-regulation of genes associated with fibrosis. Statistical significance for all comparisons was calculated using Student’s t-test between the two considered conditions with a two tailed distribution and unequal variance. * p ≤ 5 x 10^-2^, ☆ p ≤ 1 x 10^-2^, ✢ p ≤ 1 x 10^-3^.

**
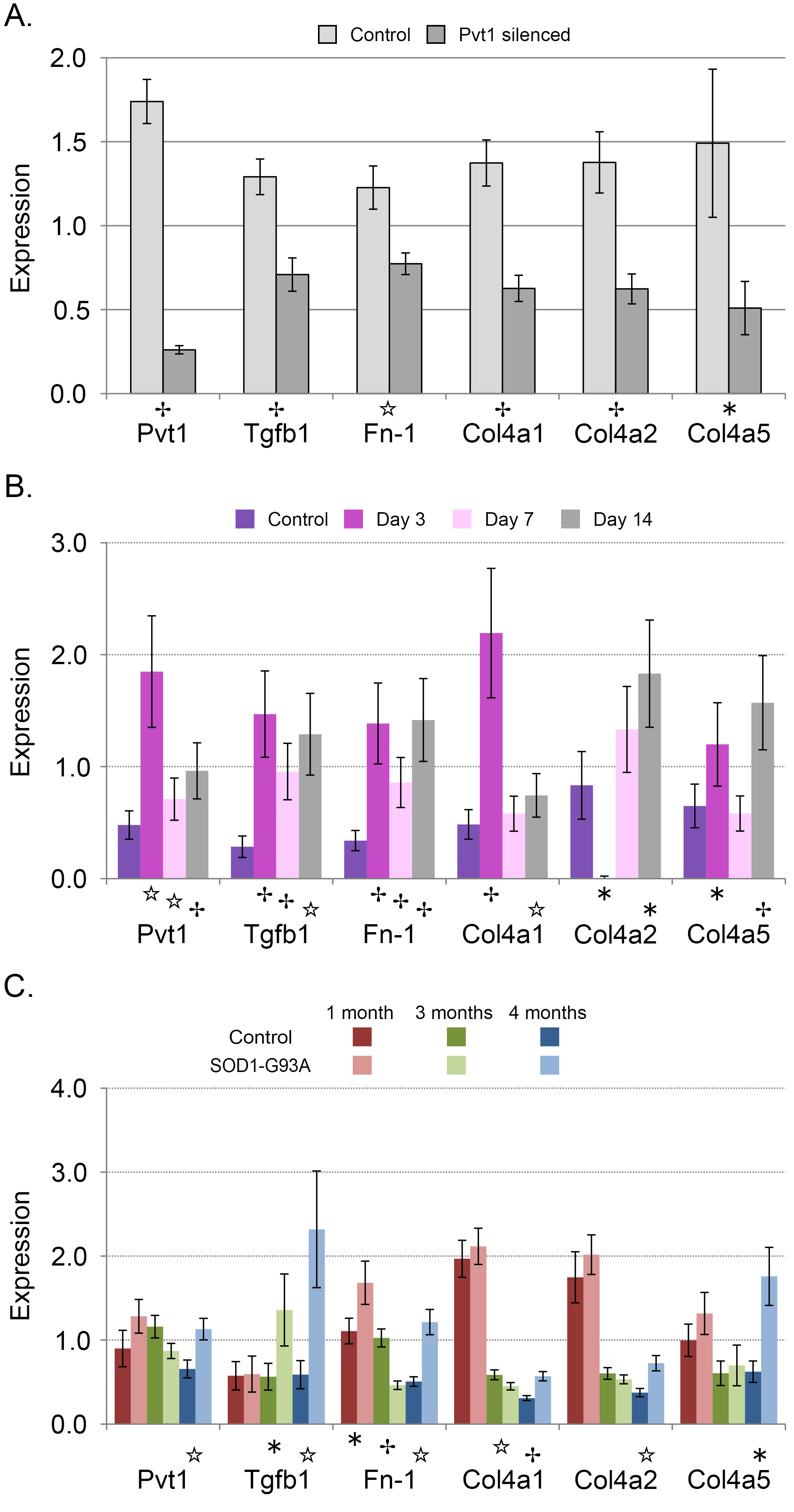
**

1. Trask, H.W., Cowper-Sal-lari, R., Sartor, M.A., Gui, J., Heath, C.V., Renuka, J., Higgins, A.J., Andrews, P., Korc, M., Moore, J.H. *et al.* (2009) Microarray analysis of cytoplasmic versus whole cell RNA reveals a considerable number of missed and false positive mRNAs. *RNA*, **15**, 1917-1928.
