# Supplementary material for: Single cell analysis reveals the involvement of the long non-coding RNA Pvt1 in the modulation of muscle atrophy and mitochondrial network
Source: Nucleic Acids Res. 2019 Jan 16;47(4):1653–70. doi: 10.1093/nar/gkz007 (PMC6393313; doi:10.1093/nar/gkz007)
Supplement: Supplementary Data [file gkz007_supplemental_files.zip › Supplemental methods-REVISION.docx]

**Single cell analysis reveals the involvement of the long non-coding RNA Pvt1 in the modulation of muscle atrophy and mitochondrial network**

Enrico Alessio^1^, Lisa Buson^1^, Francesco Chemello^1^, Caterina Peggion^2^, Francesca Grespi^1^, Paolo Martini^1^, Maria Lina Massimino^3^, Beniamina Pacchioni^1,7^, Caterina Millino^1,7^, Chiara Romualdi^1^, Alessandro Bertoli^2,4^, Luca Scorrano^1,5^, Gerolamo Lanfranchi^1,6,7^, and Stefano Cagnin^1,6,7^ *

Department, Institution, Town, State, Postcode, Country

^1^ Department of Biology, University of Padova, Padova, 35131, Italy

^2^ Department of Biomedical Sciences, University of Padova, 35131 Padova, Italy

^3^ CNR Neuroscience Institute, 35131, Padova, Italy

^4^ Padova Neuroscience Center, University of Padova, 35131, Padova, Italy

^5^ Venetian Institute of Molecular Medicine, 35131 Padova, Italy

^6^ CIR-Myo Myology Center, University of Padova, Padova, 35131, Italy

^7^ CRIBI Biotechnology Center, University of Padova, 35131, Padova, Italy

* To whom correspondence should be addressed. Tel: +39 049 827 6162; Fax: +39 049 827 6159; Email: stefano.cagnin@unipd.it

**Microarray experiments**

Fluorescent cRNA to hybridize onto microarray was produced using Low Input Quick Amp Labeling Kit (Agilent) according to manufacturer instructions. We used a different approach when experiments were performed on single myofibers. To obtain a sufficient amount of cDNA for microarray experiments, RNA purified from a single myofiber was exponentially amplified using the TransPlex Whole Transcriptome Amplification 2 Kit (Sigma-Aldrich) in accordance with the manufacturer’s instructions. To remove the residual primers and nucleotides, amplification product was purified with the GenElute PCR Clean-up columns (Sigma-Aldrich). Resulting cDNA was quantified with Nanodrop ND-1000 spectrophotometer (Celbio). 2 µg of amplified-purified cDNA were directly labelled using the Genomic DNA Enzymatic Labelling Kit (Agilent Technologies) as described by the manufacturer. Labelled cDNA was purified using the Amicon 30kDa filters (Millipore) and quantified using NanoDrop ND-1000 spectrophotometer.

800 ng of labelled sample were mixed with 5 µl of 10X Blocking Agent (Agilent Technologies) and water to a final volume of 25 µl. Samples were denatured at 95°C for 2 min and added to 25 µl of 2X GEx Hybridization Buffer HI-RPM (Agilent Technologies). 40 µl of prepared mix was dispensed onto the array. Slides were loaded into the Agilent SureHyb chambers and hybridization was performed in a hybridization oven at 65°C for 17 hours with 10 rpm rotation. Finally, slides were washed using Wash Buffer Kit (Agilent Technologies) and dried at room temperature.

**qPCR analysis**

*Primers design*. Primers were bioinformatically designed using the tool Primer3 (http://biotools.umassmed.edu/bioapps/primer3_www.cgi). Each primer couple was tested with the on-line OligoAnalyzer (Integrated DNA Technologies; https://eu.idtdna.com/calc/analyzer) for their ability to form hairpins, omo- and etero-dimers. Finally, selected primers were in silico tested to verify their amplification specificity using the tool in-Silico-PCR implemented in the UCSC Genome Browser (https://genome.ucsc.edu/cgi-bin/hgPCR). See Supplemental Table S1 for the primer list.

*Retrotranscription and qPCR.* After RNA extraction and quality analysis 1 µg of total RNA was retrotranscribed from each analyzed sample as following described. 5 µl of random examers (50 ng/µl), 1 µl of oligod(T) (500 µg/ml), 1 µl of dNTPs (10mM) were added to 1 µg of total RNA. H_2_O DNase/RNase free was used to bring the volume to 13 µl. This mix was heated to 65°C for 2 min and then transferred on ice. After cooling down the temperature 4 µl of 5X First-Strand Buffer (250 mM Tris-HCl, pH 8.3; 375 mM KCl; 15 mM MgCl2) and 2 µl of DTT (0.1 M) were added to the mix. Samples were incubated at 25°C for 2 min and then 1 µl of SuperScript® II (thermo Fisher Scientific) was added. The prepared mix was maintained at 25°C for 10 min and then heated to 42°C for 2 h. At the end of the retrotranscription the enzyme was inactivated at 65°C for 10 min.

cDNA obtained was precipitated adding 1/10 volumes of sodium acetate (3 M, pH 5.2) and 2.8 volumes of absolute ethanol, incubated at -20° C over night, then centrifuged at 13,000 x g for 20 min at 4° C. After two washes with 75% ethanol cDNA was resuspended in nuclease free water (20 µl).

The quantitative real time PCR was performed on 7500 Real-Time PCR System (Applied Biosystems) using the EvaGreen chemistry (Solis ByoDyne) and the following settings: activation step (x1) 95°C for 12 min.; PCR Cycle (x40) 95°C for 15 sec. (denaturation), 60°C for 20 sec. (annealing), 72°C for 35 sec. (elongation); final elongation (x1) 72°C for 3 min.; dissociation curve (x1).

The mix used for qPCR was prepared as follow (mix described is for one well):

| Component | Volume µl |
| --- | --- |
| H_2_O DNase/RNase *free* | 5.6 |
| *primer* *Forward* (10 µM) | 0.2 |
| *primer* *Reverse* (10 µM) | 0.2 |
| *Buffer* 5x (*HOT TAQ EvaGreen® qPCR Mix Plus, Microtech®)* | 2 |
| cDNA (5 ng/µl or 10 ng/µl for low expressed genes). | 2 |
| Total volume | 10 |

Before performing qPCR experiments primers were experimentally tested to verify their amplification efficiency. Serial dilutions from 3.2 to 0.4 ng/µl of cDNA (final concentration in 10 µl reaction volume) were used to calculate primers efficiency. In the table below are indicated results for each primer couple amplifying tested genes. cDNA was synthesized retrotranscribing, as previously described, a pool of RNAs derived from C2C12 myoblasts, *soleus*, EDL and TA skeletal muscles. Primers for Sdh and Cox2 genes were tested on DNA dilutions. Primers for low expressed genes (underlined genes in the table) were tested using higher concentrations of cDNA (from 6.4 to 0.8 ng/µl; final concentration in the reaction volume). We indicated R^2^ for the linear regression in the graph representing log_2_[cDNA] vs C_t_, the y-intercept, melt temperature (T_m_), and the efficiency of the amplification calculated as 10^-1/m^ where m is the angular coefficient of the straight line interpolating points of the graph previously described.

| **Gene** | **R^2^** | **y-intercept** | **T_m_ (°C)** | **Efficiency** |
| --- | --- | --- | --- | --- |
| B2m | 0.94 | 31.7 | 80.5 | 96% |
| Tbp | 0.98 | 28.7 | 80.5 | 99% |
| Txn1 | 0.99 | 23.3 | 67.5 | 99% |
| 1110006G14Rik | 0.98 | 34.1 | 81.0 | 99% |
| Snhg7 | 0.97 | 29.8 | 80.0 | 102% |
| Gm16062 | 0.95 | 33.4 | 80.0 | 99% |
| Cops3 | 0.97 | 26.7 | 84.5 | 102% |
| Dancr | 0.98 | 30.6 | 81.0 | 105% |
| Dio3os | 0.99 | 39.5 | 81.5 | 101% |
| Dio3 | 0.99 | 31.1 | 83.0 | 100% |
| Dleu2 | 0.99 | 35.2 | 89.5 | 124% |
| Dnm3os | 0.98 | 33.0 | 78.5 | 120% |
| Dnm3 | 0.95 | 35.3 | 81.5 | 101% |
| Gas5 | 0.99 | 30.1 | 81.5 | 101% |
| Gm6781 | 0.98 | 26.4 | 84.0 | 109% |
| Gt(ROSA)26Sor | 0.95 | 35.8 | 74.5 | 100% |
| H19 | 0.98 | 27.6 | 84.0 | 100% |

| **Gene** | **R^2^** | **y-intercept** | **T_m_ (°C)** | **Efficiency** |
| --- | --- | --- | --- | --- |
| Igf2os | 0.96 | 36.2 | 85.0 | 96% |
| Igf2 | 0.97 | 34.4 | 84.5 | 99% |
| Airn | 0.97 | 35.8 | 85.0 | 103% |
| Igf2R | 0.99 | 30.8 | 87.5 | 96% |
| Mir143hg | 0.96 | 32.0 | 85.5 | 104% |
| Mir22hg | 0.98 | 27.7 | 86.0 | 111% |
| Mirg | 0.96 | 40.1 | 78.0 | 98% |
| 2310065F04Rik | 0.98 | 31.3 | 83.0 | 104% |
| Myh3 | 0.99 | 33.3 | 87.5 | 100% |
| Nctc1_lncRNA | 0.96 | 29.7 | 80.5 | 101% |
| Nctc1_RI | 0.98 | 36.6 | 77.5 | 100% |
| Neat1 | 0.99 | 28.5 | 80.5 | 96% |
| 1110020A21Rik | 0.96 | 38.1 | 80.5 | 102% |
| Ppm1b | 0.98 | 28.9 | 78.5 | 104% |
| Pvt1 | 0.98 | 30.7 | 77.5 | 106% |
| Snhg1 | 0.99 | 30.2 | 78.5 | 99% |
| Snhg6 | 0.99 | 28.0 | 79.5 | 104% |
| Lc3 | 0.94 | 28.9 | 82.5 | 102% |
| Bnip3 | 0.97 | 31.6 | 81.0 | 103% |
| Bnip3L | 0.98 | 26.3 | 84.0 | 97% |
| ^CE^RNA | 0.99 | 36.2 | 81.5 | 99% |
| Linc-MD1 | 0.98 | 32.3 | 84.0 | 100% |

| **Gene** | **R^2^** | **y-intercept** | **T_m_ (°C)** | **Efficiency** |
| --- | --- | --- | --- | --- |
| Munc | 0.97 | 35.8 | 80.5 | 101% |
| Pink1 | 0.96 | 30.2 | 82.5 | 100% |
| Myh1 | 1.00 | 27.6 | 87.5 | 101% |
| Myh2 | 0.99 | 28.7 | 80.5 | 104% |
| Myh4 | 0.97 | 33.7 | 89.5 | 102% |
| Myh7 | 0.97 | 34.4 | 89.0 | 102% |
| Opa1 | 0.99 | 29.1 | 81.0 | 105% |
| Mfn1 | 0.97 | 28.1 | 81.0 | 104% |
| Mfn2 | 0.98 | 27.1 | 86.0 | 100% |
| Drp1 | 0.99 | 28.7 | 80.5 | 107% |
| Fis1 | 0.96 | 26.3 | 87.0 | 105% |
| Plin1 | 0.96 | 35.2 | 82.0 | 102% |
| Plin2 | 0.99 | 24.7 | 82.5 | 114% |
| Cpt1a | 0.98 | 33.2 | 87.0 | 105% |
| Cpt1b | 0.98 | 31.5 | 83.0 | 101% |
| Lipe | 0.97 | 32.0 | 85.5 | 101% |
| Atp5d | 0.98 | 24.9 | 85.5 | 102% |
| c-Myc | 0.99 | 33.8 | 81.5 | 97% |
| Bcl-2 | 0.98 | 32.9 | 81.0 | 101% |
| Bax | 0.99 | 26.3 | 84.5 | 110% |
| Bak1 | 0.99 | 26.4 | 82.0 | 110% |
| Beclin 1 | 0.99 | 26.9 | 84.5 | 109% |

| **Gene** | **R^2^** | **y-intercept** | **T_m_ (°C)** | **Efficiency** |
| --- | --- | --- | --- | --- |
| Cox2 | 1.00 | 30.4 | 80.0 | 100% |
| Sdh | 0.98 | 27.2 | 83.5 | 102% |
| Pax7 | 0.99 | 32.5 | 80.0 | 114% |
| CD56 | 0.99 | 27.6 | 78.5 | 104% |
| Myf5 | 0.98 | 28.6 | 74.5 | 103% |
| Myod1 | 0.99 | 27.3 | 80.5 | 102% |
| Mrf4 | 0.96 | 32.8 | 80.5 | 103% |
| Tgfb1 | 0.98 | 29.6 | 82.0 | 99% |
| Fn-1 | 0.98 | 23.7 | 85.0 | 95% |
| Col4a1 | 0.98 | 26.5 | 84.0 | 96% |
| Col4a2 | 0.97 | 27.2 | 77.0 | 104% |
| Col4a5 | 0.97 | 32.2 | 81.0 | 98% |

**Fluorescence in situ hybridization**

*Probes construction.* cDNA was used to produce, by PCR (see supplemental Table S2 for the list of primers), an amplicon of ~600 bases matching the chosen lncRNAs. Amplicons were cloned into pSC-A-amp/kan plasmid using StrataClone PCR Cloning Kit (Agilent Technologies). Plasmids were amplified, purified (PureLink MiniPrep kit; ThermoFisher Scientific) and the sequences were analyzed with Sanger sequencing (Supplemental Table S2).

Plasmids were linearized using a restriction enzyme to allow *in vitro* transcription for the production of the Fluorescence In Situ Hybridization (FISH) probes. Plasmid restriction with HindIII (New England Biolabs) was used before *in vitro* transcription with T7 RNA polymerase (New England Biolabs) while SmaI was used in association with T3 RNA polymerase *in vitro* transcription (New England Biolabs). When the regions cloned presented a restriction site for HindIII or SmaI, the next closest option was used in order to maintain the integrity of the inserted sequence. Both the antisense and the sense sequences were synthesized to produce both matching and non-matching RNA probes. In vitro transcription was performed adding Aminoallyl-UTP (Thermo Fisher Scientific) to label probes using Cy3 Mono-Reactive Dye (GE Healthcare Life Sciences). Labelled probes were fragmented to a final size comprised between 50 and 150 base pairs using RNA Fragmentation Reagents (Thermo Fisher Scientific).

*FISH Experiment.* FISH experiments were performed on both proliferating and differentiating C2C12 cells and on 20 µm thick sections of TA.

C2C12 myoblasts were cultured on glass coverslips while fresh excised muscles were included in Optimal Cutting Temperature (OCT) compound (VWR International) and flash frozen in liquid nitrogen. Each sample was fixed in 1x Phosphate-Buffered Saline (PBS, Thermo Fisher Scientific) with 4% Paraformaldehyde (PFA, Sigma-Aldrich) for 7 min at room temperature and permeabilized in water with 70% (v/v) ethanol overnight at -20°C.

Fragmented probes were diluted in enough FISH hybridization solution (30% Formamide, 5X SSPE, 4X Denhardt’s Solution, 100 µg/ml salmon sperm DNA, 100 µg/ml tRNA, 5 mM DTT, 4 µl Rnase Inhibitor 20 U/µl) to cover the sample. 1 μg of probe was used to treat each C2C12 culture coverslip or muscle sections. Hybridization was performed overnight in a humid environment at 32°C. Samples were then washed for 5 min twice with SSC 3X at 30°C; twice with Wash Buffer II (WB-II: 10 mM Tris HCl pH 7.6, 0.5 M NaCl, 0.1% Tween-20) at 30°C; and treated with RNaseA in WB-II for 45 min at 37°C to remove excess of probe; twice in WB-II at 37°C; twice in SSC 2X at Room Temperature (RT) for 5 min; twice in SSC 1X at RT for 5 min. Nuclei were stained using DAPI and then samples where mounted using Fluoromount mounting medium (Sigma-Aldrich) to preserve the fluorescence.

*Image Acquisition and Analysis.* Images were acquired with Leica TCS SP5 confocal laser microscope. When comparing fluorescence levels was necessary, images were acquired during the same session, using the same microscope parameters.

**Pvt1 down-expression**

*In vitro silencing. In vitro* Pvt1 silencing experiments were performed by transfecting proliferating C2C12 myoblasts with Lipofectamine® 2000 Transfection Reagent (Termo Fisher Scientific) and antisense LNA™ GapmeRs (Exiqon) (Pvt1_1 ACCGTAGTAGAGTTAA; Pvt1_3 AGTCAACGCTTCACAT). Cells transfected with Lipofectamine® 2000 and Antisense LNA™ GapmeR Negative Controls (Exiqon) were used as negative controls.

C2C12 myoblasts were detached prior to transfection and seeded at high density on a 24 multiwell plate, in 500 µl of Opti-MEM® medium (Thermo Fisher Scientific) with 10% FBS, 35 pmol of GapmeRs and 3 µl of Lipofectamine® 2000. After 48 h, transfection medium was removed. To analyse the effects of Pvt1 silencing, cells were detached with Trizol right after removing the transfection medium. Silencing efficiency was assessed analyzing the expression of Pvt1 using qPCR.
