# Supplementary material for: Single cell analysis reveals the involvement of the long non-coding RNA Pvt1 in the modulation of muscle atrophy and mitochondrial network
Source: Nucleic Acids Res. 2019 Jan 16;47(4):1653–70. doi: 10.1093/nar/gkz007 (PMC6393313; doi:10.1093/nar/gkz007)
Supplement: Supplementary Data [file gkz007_supplemental_files.zip › Table S1-REVISION.docx]

**Table S1. Primers used in qRT-PCRs**

| **Reference genes** | | |
| --- | --- | --- |
| **Gene** | **Primer** | **Sequence** |
| B2m | For | CCGTCTACTGGGATCGACAC |
| B2m | Rev | GCTATTTCTTTCTGCGTGCAT |
| Tbp | For | TGCTGTTGGTGATTGTTGGT |
| Tbp | Rev | AACTGGCTTGTGTGGGAAAG |
| Tnx1 | For | TCCAATGTGGTGTTCCTTGA |
| Tnx1 | Rev | GGCTTCAAGCTTTTCCTTGTT |
| **LncRNAs and coding genes** | | |
| 1110006G14Rik | For | TCGGTTTGTGTACTGCTGAGA |
| 1110006G14Rik | Rev | GGGACTCCAGGATCTTCGTA |
| Snhg7 | For | GATTGGCTTGGGTTTGACTCT |
| Snhg7 | Rev | GGGTCCTCCATCCAGTGTTT |
| Gm16062 | For | CCCTGTCCCTACCGAAGAG |
| Gm16062 | Rev | GTCCCAAGAACTCCCAGAATC |
| Cops3 | For | ATTCAACCAACAACCCGTCT |
| Cops3 | Rev | AGTGAAGGTCTCGCTGTGCT |
| Dancr | For | CCCTTCTTCATGTCCCACTG |
| Dancr | Rev | AAACGGCGAGCATGTCATAG |
| Dio3os | For | TCCTTCTGGTCCTGCTCTATG |
| Dio3os | Rev | TTTCCCAAGTGTCCCATATCA |
| Dio3 | For | CATCCGCAAGCATTTCC |
| Dio3 | Rev | GCATCTCCTCGCCTTCAC |
| Dleu2 | For | GATGTTGGGGCGGAGAG |
| Dleu2 | Rev | TCAGCGACGGAGGAAGAC |
| Dnm3os | For | GGCACACTTGAGATTTTATCCTTC |
| Dnm3os | Rev | CGACTACTACTGTTTGTTGTTTCCA |
| Dnm3 | For | CCAAACCAGAAACCCTACCA |
| Dnm3 | Rev | CGGGGAGAGAAGAGGAATACA |
| Gas5 | For | CTGGCTTGCTTGGGTAAGA |
| Gas5 | Rev | GCATGTTCAAAAGCTAAATGTTATG |
| Gm6781 | For | GGAGGTGTGGGGCAGATAC |
| Gm6781 | Rev | GCTGTAGGGCGTTGGTTATG |
| Gt(ROSA)26Sor | For | TTTGTATGTGAGGATAAAGGTGTTTG |
| Gt(ROSA)26Sor | Rev | TTTGGAACTTTGGGGAAATGT |
| H19 | For | GAGACCACCACCCACATCAT |
| H19 | Rev | GGAGGAAGAAGAAAAAGACAGGA |
| Igf2os | For | GCTGGAAGGGGCTGAGTC |
| Igf2os | Rev | CAGTGGTGGAGAGCAGAAGC |
| Igf2 | For | CGGTGTGTGTCAGCCAAG |
| Igf2 | Rev | TGAGTTTCTGTCCAATGTTCCA |
| Airn | For | CTCCAGAAACTAGACACTACAGACCA |
| Airn | Rev | TGTAAAGTCTTCAAGGATCTAGGGC |
| **Gene** | **Primer** | **Sequence** |
| Igf2R | For | GTTGGTGTAGGGCCAGTGTT |
| Igf2R | Rev | AAATTCTGCGGGGTACTTTG |
| Mir143hg | For | CAGTCACCACGAAGCAAAGG |
| Mir143hg | Rev | ACCCCCAAACCACCTACTCA |
| Mir22hg | For | ATGGCTCTGCTGTCCTCATC |
| Mir22hg | Rev | GGTCCTCCACTGTCTTGTGC |
| Mirg | For | CCACCCCAGAGCCTTGTATT |
| Mirg | Rev | AAGAGCAGAAACCCCTCCTTC |
| 2310065F04Rik | For | GTGAGCGTGAGATGTTCGTG |
| 2310065F04Rik | Rev | AAAGTGCATCCCCTCTGCTT |
| Myh3 | For | CGCCTGAAGAAGAAGATGG |
| Myh3 | Rev | CGGAGGTGCTTGATGGT |
| Nctc1_lncRNA | For | TGAGAAGGAAGCAGTGACAAGTA |
| Nctc1_lncRNA | Rev | AAGGCAGAGTTGGGCTATCC |
| Nctc1_RI | For | CAGCCCAGAGTTTTGTAAGCC |
| Nctc1_RI | Rev | CAAATAACACCTGGAGAGGAAGG |
| Neat1 | For | CATTGTGGGTTTGGCTTGA |
| Neat1 | Rev | GACAGTTTAACAGCTTCCCCTCT |
| 1110020A21Rik | For | GGTAGAGTCCTCCGTCAGTCA |
| 1110020A21Rik | Rev | GCCATTTGAACCAATCAGAAA |
| Ppm1b | For | AGGGTTAGAGATTACTGGTTGGA |
| Ppm1b | Rev | GGTATGAGGATGGAAAATAAGCA |
| Pvt1 | For | CTTGTATGGGTGGTGGCTTT |
| Pvt1 | Rev | CCAGGGAGAGAGTGGTGTG |
| Snhg1 | For | AGGGATCATTTTTGTGCGTAGA |
| Snhg1 | Rev | GCTCATTCTTTTCCTCAGACCTG |
| Snhg6 | For | ATGTAGGTGGCTGTAGTGGATG |
| Snhg6 | Rev | CAATAAAGGGTTTTTAGACATCACAG |
| ^CE^RNA | For | GCCAAGTATCCTCCTCCAGC |
| ^CE^RNA | Rev | AAGCTGAGCACTCTGGGAGA |
| Linc-MD1 | For | CCCAGGTTCTATGCTCCTCA |
| Linc-MD1 | Rev | GTCCCTCCTCCTCTGTGGTT |
| Munc | For | GCAGAGGCAGCGTGAGAG |
| Munc | Rev | AGTCATTCTATTCCAGCAAACCTG |
| **Coding genes involved in mito/auto-phagy** | | |
| Lc3 | For | TGGGAGTTCTGGTCAGGTTC |
| Lc3 | Rev | CCTAATCCACTGGGGACTGA |
| Bnip3 | For | GCTTTGCAGGATGAGGATTC |
| Bnip3 | Rev | ACAAAACTGACCACCCAAGG |
| Bnip3L | For | GAGCAGCTCAAGAGGCAGTT |
| Bnip3L | Rev | GTCCCTGCTGGTATGCATCT |
| Pink1 | For | GAGAAGAGCTGCGTGGAGAC |
| Pink1 | Rev | GCCTCACACTCCAGGTTAGC |

| **Coding genes for myofiber specification** | | |
| --- | --- | --- |
| **Gene** | **Primer** | **Sequence** |
| Myh1 | For | ACCTTGTGGACAAACTGCAA |
| Myh1 | Rev | AGCTTGTTGACCTGGGACTC |
| Myh2 | For | TTGGTGGATAAACTCCAGGC |
| Myh2 | Rev | CAGCTTGTTGACCTGGGACT |
| Myh4 | For | TAGGGTGAGGGAGCTTGAAA |
| Myh4 | Rev | GTTTGTCCACCAAGTCCTGC |
| Myh7 | For | AGCAGGAGCTGATTGAGACC |
| Myh7 | Rev | TGTGATAGCCTTCTTGGCCT |
| **Coding genes for mitochondria dynamics** | | |
| Opa1 | For | TGCAGGTTCACCTGGAGAAAC |
| Opa1 | Rev | TGTCTGACACCTTCCTGTAATGCTTGT |
| Mfn1 | For | CCTGTCTTCACAATAGGCACA |
| Mfn1 | Rev | CCCTGTCTCAAAACTAATCAACC |
| Mfn2 | For | CATTCTTGTGGTCGGAGGAG |
| Mfn2 | Rev | AAGGAGAGGGCGATGAGTCT |
| Drp1 | For | AACCTGACACTTGTGGATTTACC |
| Drp1 | Rev | TCTGAAGCTCGATGTCCTTG |
| Fis1 | For | TGGTGTCTGTGGAGGATCTG |
| Fis1 | Rev | AAATTGCGTGCTCTTGGAC |
| **Coding genes for lipid droplets, FA catabolism and ATP synthesis** | | |
| Plin1 | For | AACGTGGTAGACACTGTGGTACA |
| Plin1 | Rev | TCTCGGAATTCGCTCTCG |
| Plin2 | For | CTCCACTCCACTGTCCACCT |
| Plin2 | Rev | GCTTATCCTGAGCACCCTGA |
| Cpt1a | For | GACTCCGCTCGCTCATTC |
| Cpt1a | Rev | TCTGCCATCTTGAGTGGTGA |
| Cpt1b | For | GAGTGACTGGTGGGAAGAATATG |
| Cpt1b | Rev | GCTGCTTGCACATTTGTGTT |
| Lipe | For | CTTCCTGCAAGAGTATGTCACG |
| Lipe | Rev | ATGGCAGGTGTGAACTGGA |
| Atp5d | For | AGACGGCACCACGACTAAGTA |
| Atp5d | Rev | GTGTCACAGCTTCTTCAGCTAGTAA |
| **Genomic regions for the identification of nuclear and mitochondrial DNA** | | |
| Cox2 | For | CTACAAGACGCCACAT |
| Cox2 | Rev | GAGGGGGAGAGCAAT |
| Sdh | For | TACTACAGCCCCAAGTCT |
| Sdh | Rev | TGGACCCATCTTCTATGC |
| **Pvt1 mechanism** | | |
| c-Myc | For | TCCTCGGATTCTCTGCTCTC |
| c-Myc | Rev | CTCTGACCTTTTGCCAGGAG |
| Bcl-2 | For | CACATGCTCTTTGTGAATTGCT |
| Bcl-2 | Rev | CTTCTCTCCTTCCGCCAAGT |

| **Gene** | **Primer** | **Sequence** |
| --- | --- | --- |
| Bax | For | TTTGCTACAGGGTTTCATCCA |
| Bax | Rev | GCTCCAAGGTCAGCTCAGG |
| Bak1 | For | AGTGAGGGCAGAGGTGAGAG |
| Bak1 | Rev | GGAGAGCCTGATGGATGTGT |
| Beclin 1 | For | CAATGCCACCTTCCACATC |
| Beclin 1 | Rev | CATTCCACTCCACAGGAACAC |
| **Markers of satellite cells** | | |
| Pax7 | For | CACCTGAGTCTTCGTTCACCT |
| Pax7 | Rev | CTGTGGATGTCACCTGCTTG |
| CD56 | For | AGTGTCAGTGTCGGTGTGTCT |
| CD56 | Rev | GCCATGCTGGTCTTTATGTG |
| Myf5 | For | CTGCTCTGAGCCCACCAG |
| Myf5 | Rev | GACAGGGCTGTTACATTCAGG |
| Myod1 | For | GACACTCTTCCCAACTGTCCTT |
| Myod1 | Rev | AGACTTCTGCTCTTCCCTTCC |
| Mrf4 | For | GGAGGAGGTGGTGGAGAAGT |
| Mrf4 | Rev | TTCCTGCTGGGTGAAGAATG |
| **Genes involved in the fibrosis** | | |
| Tgfb1 | For | GGAGGAGGAGTGGGAGGA |
| Tgfb1 | Rev | GTAGCAGCGGAAAAGTCTCAA |
| Fn-1 | For | GCTTTCTTTTGCCATCTGACCT |
| Fn-1 | Rev | AGTCCTGTGGGAGGGGTGT |
| Col4a1 | For | CAAAGCCAAACCCATTCC |
| Col4a1 | Rev | GCAAACCCTGAAAATACTCCA |
| Col4a2 | For | GGTTTCTTGGAGGAGGAGATG |
| Col4a2 | Rev | GGGAGGTGGCAGTTATGGA |
| Col4a5 | For | TCACTCACTCTCCCCACCTT |
| Col4a5 | Rev | GCTAGTCAGCATGTTCTCTTCGT |
