# Supplementary material for: Single cell analysis reveals the involvement of the long non-coding RNA Pvt1 in the modulation of muscle atrophy and mitochondrial network
Source: Nucleic Acids Res. 2019 Jan 16;47(4):1653–70. doi: 10.1093/nar/gkz007 (PMC6393313; doi:10.1093/nar/gkz007)
Supplement: Supplementary Data [file gkz007_supplemental_files.zip › Table S2.docx]

**Table S2. Probes for FISH experiments.** Primers used for the construction of probes by PCR amplification of cDNA are listed in the table in association with sequence cloned in pSC-A-amp/kan plasmid. Plasmids were used for *in vitro* transcription to produce labeled RNA. RI in the Nctc1 name indicates Retained Intron isoform.

| **Target Gene** | **Description** | **Sequence** |
| --- | --- | --- |
| Neat1 | For | TCCAATGCTGCTATCTAAAGG |
| Neat1 | Rev | GTAAAGGGGAGGAAAATGGT |
| Neat1 | Probe | ATAAAAGGGGATATGCCCTTATACACACATACAGGAATTTAATGTGTGAGCCATGCCTTCAAAACACATCTTGAATTTCTGGAATTGGCCAGAAGACAACAGGGTTTGCTTATTTAACCTTTCAAATCAGTCATTGTGACAAGGGGACAGGGTCTTGCGCTTGCTATGAGATCATTGTGATGCTTGTAGAAACCTCATGGGTGCTTTGCCACTGAATACATCCATTGTGGGTTTGGCTTGAATGGTGCTTAAAAACCATCCCTGAGCAGAGGGGAAGCTGTTAAACTGTCAGCCAAAGCAGTTTGGGAAATAAAAGAGACTGGGCCCTGGGTCATCTTACTAGATAACACTTTGTAAAAATTGGTTCTGAAAACCCTGTTTATTTGCATATTTGTGAAAACCCTGTATATGTGGTTGTTTTGTGAGTGTGCTTAAAAGTGGTGTGACCAGGGCAAGATCGCTCATTGGAACAGCTGTGTGGAATGGGGAAATGTGAAGAAAGCTGTAACTGCAGGTTGGTCGCACACGCTTCTCTGTACTAAAGTGTTCAGTGTACAAACCCACTAACCATTTTCCTCCCCTTTACA |
| Airn | For | TCAGCACAAGTCAGCAGGAG |
| Airn | Rev | TTAGGTTGAAGGGTTCTTTGGA |
| Airn | Probe | TTAGGTTGAAGGGTTCTTTGGAAAGTCAGTTGTGTTGGATGGTGTTTTGCTGGGACAAACACATGAAGGAGTTTTTCCCTGAAGTGGACACAGGTGAAAGGGTGTTTTGCTAAGGCAGACTTGTGAAGGAATGTTTCACTGAAGCAGACACGCGTGAAAGGATTTTCTGCTAAAGCAAGCACGTGAAAGGACACATGGTGACGGATTCTTCATTTACAACACACAAGTATTGGTCTGCCTTAAATTGTGTAGTTGAGCGCCATTTGTCAGGACTCCAGAAATCGAAATACACCAAAAAAACCTTCTGGTAGTATGCTGCGGCTTCTTGCAGCTTCTGAGGACTTGGGTCAACTGGCAGAGTGATGTTAGCTGATATAGACTCGTGGAGTTTTGCTAAGACAGACTCACATGCTGAGGCAAGAGCTGTGGAGAGCACGTGATTTTTGGAGGGAGTGTAAATAGGACTGAGAGGGGCTTGCTTGTAGAGCTAGCTCTGCAATACTTCTTGGTCTCTCATCTTTGATGATCTTTGCTTCACTGAGAGAGGCACAGCAAAGAACTCCTGGTGTCCCTGTTGGTTCTGGTCCCTCCTGCTGACTTGTGCTGA |

| **Target Gene** | **Description** | **Sequence** |
| --- | --- | --- |
| H19 | For | CCTCCCCCTACCTTGAACC |
| H19 | Rev | CAGACGGAGATGGACGACA |
| H19 | Probe | CAGACGGAGATGGACGACAGGTGGGTACTGGGGCAGCATTGCCAAAGAGGTTTACACACTCGCTGTATACATTCATACGGAGAGACTCAAAGCTATCTCCGGGACTCCAAACCAGTGCAATCGACTTAGTGCAAATTCAAAAGGGGGTAAATGGGGAAACAGAGTCACGGGTGCTTTGAGTCTCTCAAGCAAGGAAGGAACAAAAGGAAAGAAACAGAATTACAGGAAAGGAGGAAGAAGAAAAAGACAGGAGGGAGATGATGAAGTCATCCCGGGGTAGAGGCTTGGCTCCAGGATGATGTGGGTGGTGGTCTCCCGGGTCAGGCAGAGTTGGCCATGAAGATGGATTCTCAGGGGTGGGTGGGTGCTATGAGTCTGCTCTTTCAAAATGTTGACACCATCTGTTCTTTCAGCTTCACCTTGGAGCAGATTCCTGGGGCAGGTAGTGTAGTGGTTCTGATTGCAGCATCTTCTTGATTCAGAACGAGACGGACTTAAAGAAGTCCCCGGATTCAAAGGCCCAGACATGAGCTGGGTAGCACCATTTCTTTCATCTTGAGGGTTCAAGGTAGGGGGAGG |
| Mir143hg | For | AAGCAATAACACCCACAACC |
| Mir143hg | Rev | TAGAACCTGCCGATGACTTT |
| Mir143hg | Probe | AAGCAATAACACCCACAACCACACCCCAAATAGTCTGGGCCTCCACCTCCGAAGATTCATCCCTGGACCAGTCACCAGTCAAGGCAAGAGTGATGGCTGGTAGGATTCCTCCTACACTGGAGAAGAGCCCCAGAGAGGAACCTTCCTGAGAGGCCGAAAAGGGACAGCAACAGCCGCCCAGCTCCCAGATCTCGTCCCCTCCTCTCACCCCTCCCGGGAGCTTCCCAACGGACAGAAGGGAGAGATCCCCTTGCCTGGAGACATCTTCTGAAGACATGGGAAAAGGAGACGAGGCGGGCTACTCCCTGGCAGCAGGCCTTCAAGCTGTTGACAACCTGTACCAAGAAGAGTGCCAGCCCTGAGGAAAGGCTGCCTACCCTCTTCAGAAAACCCTGAAGGTATGTATCATCCATCACCAAGGCCACCGCAGTCACCACGAAGCAAAGGTTATCTGGGGACCTTGGGGGAGGGGGGTGTCTTAGCAAGAGCAGGGTGAGTAGGTGGTTTGGGGGTCAGGGGTGTTCTGTTCACCCCGAAGTTCCCACACAAGCCGTGGCCACAGAAGATGGTCACCACGTGTCTTCTTTTGTCTGTAGTCACATGCAAAGTCATCGGCAGGTTCTA |

| **Target Gene** | **Description** | **Sequence** |
| --- | --- | --- |
| Gt(ROSA)26Sor | For | CAGGACAGTGCTTGTTTAAGG |
| Gt(ROSA)26Sor | Rev | CTCGCACCAACACAAAAGT |
| Gt(ROSA)26Sor | Probe | CAGGACAGTGCTTGTTTAAGGCTATATTTCTGCTGTCTGAGCAGCAACAGGTCTTCGAGATCAACATGATGTTCATAATCCCAAGATGTTGCCATTTATGTTCTCAGAAGCAAGCAGAGGCATGATGGTCAGTGACAGTAATGTCACTGTGTTAAATGTTGCTATGCAGTTTGGATTTTTCTAATGTAGTGTAGGTAGAACATATGTGTTCTGTATGAATTAAACTCTTAAGTTACACCTTGTATAATCCATGCAATGTGTTATGCAATTACCATTTTAAGTATTGTAGCTTTCTTTGTATGTGAGGATAAAGGTGTTTGTCATAAAATGTTTTGAACATTTCCCCAAAGTTCCAAATTATAAAACCACAACGTTAGAACTTATTTATGAGCAATGGTTGTAGTTTCATGCTTTTAAAATGCTTAATTATTCAATTAACACCGTTTGTGTTATAATATATATAAAACTGACATGTAGAAGTGTTTGTCCAGAACATTTCTTAAATGTATACTGTCTTTAGAGAGTTTAATATAGCATGTCTTTTGCAACATACTAACTTTTGTGTTGGTGCGAG |
| Mir22hg | For | CGCTGGGAAGAGACAGAG |
| Mir22hg | Rev | ACCTACCAACTGAGCTACAACC |
| Mir22hg | Probe | CGCTGGGAAGAGACAGAGCGGTCGGCCGTGCGGACAGGTCGCAGTGATTTTGCTCCTCTGTCCACAGCAACCCCCGCACCCAGCATCAGGAACCTGTGCCTCCCACACCCTCACCTGGCTGGGCCGCAGTAGTTCTTCAGTGGCAAGCTTTATGTCCCGACCCAGCTAAAGCTGCCAGTTGAAGAACTGTTGCCCTCTGCCCCTGGCTTCGTGGAGGAAGAGGAGAAGCAGCAGCTTTGCCTATCATCCGGAAGTGTGTCCCTCCAGCACTGGGTACATGGCTCTGCTGTCCTCATCCAACATGGAGCCTCAGAGGTGAGAAGGGGCAGCCTGGAAGCAACAGAGGCAGGCACAAGACAGTGGAGGACCTGGCCTGGAACCACAAGGGCCTATCCGGTCATTGGTCAGAGAGGCACGTAGAAGCCTGGAGAACACCAGGAAAGAGAGCAGCCAGCCAGCCTCAGTGAAAGACACGTGCTTCCAGCCATCTCCTCTCAGGACCTGCCTTCCTGGGAGATGAAGGGCCTCCAGGAAGTATGGTCCCATCTCTGCCCTGCAGTTTCTATAAACAGCCTCAAGGAGCATGAGCCACCTCTGAAAGGAAATACACAGCACATTCAAAAAGAGATTCAAATGTGTAACACTGTGGGAAAACATATCTATGACTGGGGTTGTAGCTCAGTTGGTAGGT |

| **Target Gene** | **Description** | **Sequence** |
| --- | --- | --- |
| Pvt1 | For | CTTAGTGAATGCTGGCTTGTG |
| Pvt1 | Rev | TGGCTACTGAAAGAAGGAAGGT |
| Pvt1 | Probe | TGGCTACTGAAAGAAGGAAGGTAGGTCAGACAGAAGCAGGAAGATTAAAGTAGCAGAGTGGTTCAAAGGCAAGACCCCCTTTTCTGGGGTGTTTCTGACAGAAAGGATAAGAAGTGAGTCGGAAGGGCACACATTCAGCAGAAACTTTCCAAAAGATTGGTTTGTTTGTTTTTAAGACATACTGGTTGGTTCTTTAATGATCAAGAACTATTTTGAAATAATGTCTACTTGTTGGCCAACTTCAAATGACCTCTCTAAAGGCTGGCCATTGCCATACCAGAAAAGAATATCCCAAATCACACTCGCTCCTTCACACTCGCCCAGCAGCCTGGTCCCTCACTACACAGAAAGGACAGAGAGTCTGAGCATCCCTTTAGCTCTGATGAGAAAGAAGGGAGGAGGTGCAAAAAAGAAAAAGGCCACCATATGGAAAGACCCTTTCAAAAATGAGCTTCAGAAATTCTAGAGCCTTCAGGAAGGCTTGCCCAGAGAGACTTGAATCTATTTGCATGAATTGCTTTGGGTATTTTGGTCATCTACTAACACGATTTGATGATCACAGCCCCTTCTTTTTCCACAAGCCAGCATTCACTAAG |
| Nctc1_RI | For | CTTTGAGGGTCTGGGAAGGT |
| Nctc1_RI | Rev | TGCCTGGTGTGTGTTCTCTC |
| Nctc1_RI | Probe | TGCCTGGTGTGTGTTCTCTCCAGGCTGCCTGGCTTACCTTTGCTTAGAGCTAAGCAGGCCTGCTTGGGACCTCTCTGGGCCTGAGTTGTCCCGTCTGTGGAGACTGGTAATGGCTGGGGCCTGCTGTTTTCCTCTCTTGGTAATTGAAGGGGCCACTTGGGAAATCTACTCTCAAGCTTGTCTGTTGGGTCATTTTGATTTGGGGATTTCCAAACATTGGGGCCATGAAAGGTCACCCCGCTGACCCCAGCCTGCCTCATTATGTCACAGCCAGATTGCTGTGGTACCTGATGGAATCCAGAAGGCCCACACCAGTGGGAGGGCAGGCAGGCAGGCAGGCAGGCAGACGACTGAGGTGTGGGAGCAGGGCACTAGGCCCAGCTCATCCCAATTCTAAGCAAAGCTGCCTTTCCAGTCTCCATCATTTCAGGAATATCAGCCTTGGTACCACAAGTCCAGGCTGTGTGTGCCTTGCCAGCTTTGCCCTTGGGCATCAGGCGTCTGCTCCTTGTTCTGGCTTCCTTGAATGGACTTTGTCCCTTGTCCAGTCTTTCACAAGTGTCTTTACCTCCTTCTCCACTGCTGTTGGATAAATAGCTGCCTTCATCCTAGTCCACTGGTCCCTAGCCCTCCCACAAGCACCTTGTCACCTTCCCAGACCCTCAAAG |
